# The Impact of Toll‐Like Receptor 5 on Liver Function in Age‐Related Metabolic Disorders

**DOI:** 10.1111/acel.70009

**Published:** 2025-02-17

**Authors:** Dong‐Hyun Kim, Hye Sun Go, Eun Jae Jeon, Thi Quynh Trang Nguyen, Da Yeon Kim, Hansung Park, Hyo‐Ji Eom, Sung Young Kim, Sang Chul Park, Kyung A Cho

**Affiliations:** ^1^ MediSpan, Inc Bundang‐gu Gyeonggi‐do Republic of Korea; ^2^ Department of Biochemistry Chonnam National University Medical School Hwasun‐gun Jeonnam‐do Republic of Korea; ^3^ Center for Creative Biomedical Scientists Chonnam National University Medical School Hwasun‐gun Jeonnam‐do Republic of Korea; ^4^ Department of Biochemistry Konkuk University School of Medicine Seoul South Korea; ^5^ Future Life and Society Research Center Chonnam National University Medical School Hwasun‐gun Jeonnam‐do Republic of Korea

**Keywords:** aging, fibrosis, hepatic metabolism, hepatocytes, metabolic syndrome, stellate cells, toll‐like receptor 5

## Abstract

Toll‐like receptor 5 (TLR5) plays a critical role beyond its traditional function in innate immunity, significantly impacting metabolic regulation and liver health. Previously, we reported that TLR5 activation extends the healthspan and lifespan of aging mice. This study demonstrates that TLR5 deficiency leads to pronounced metabolic abnormalities with age, primarily affecting liver metabolic functions rather than intestinal inflammation. Comprehensive RNA sequencing analysis revealed that TLR5 deficiency induces gene expression changes in liver tissue similar to those caused by the methionine–choline deficient (MCD) diet, particularly affecting lipid metabolism and circadian rhythm‐related genes. TLR5 knockout (TLR5 KO) mice displayed an increased propensity for liver fibrosis and lipid accumulation under the MCD diet, exacerbating liver pathology. Both hepatocytes and hepatic stellate cells in TLR5 KO mice were functionally impacted, leading to metabolic dysfunction and fibrosis. These findings suggest that TLR5 could be a significant target for addressing metabolic diseases that arise and worsen with aging. Furthermore, understanding the mechanisms by which TLR5 activation extends healthspan could provide valuable insights into therapeutic strategies for enhancing longevity and managing age‐related metabolic disorders.

## Graphical abstract

Toll‐like receptor 5 (TLR5) deficiency accelerates age‐related metabolic disorders, leading to obesity, fatty liver, and cellular changes such as lipid accumulation in PMHs (hepatocytes) and activation of HSCs (hepatic stellate cells), which drive fibrosis. Activation of TLR5 using flagellin reverses these effects, reducing steatosis and fibrosis, and restoring cellular homeostasis in liver disease models. This study underscores the potential of TLR5‐targeted therapies for mitigating age‐related liver dysfunction and metabolic diseases.

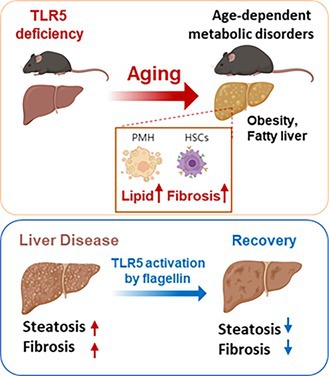

## 1 Introduction

Geroscience is an emerging field that seeks to understand the biological underpinnings of aging and its relationship with age‐related diseases. One of the critical goals of geroscience is to identify geroprotectors—substances that can slow down the aging process and mitigate the onset of age‐associated diseases. These agents are particularly crucial for targeting immunosenescence, the gradual deterioration of the immune system that occurs with aging, leading to increased susceptibility to infections, chronic inflammation, and reduced efficacy of vaccinations (Franceschi et al. 2018). The identification and development of geroprotectors that can effectively modulate immune function in the elderly are, therefore, of paramount importance. The recent COVID‐19 pandemic has underscored the critical need to address immunosenescence. Older adults have been disproportionately affected by severe outcomes from SARS‐CoV‐2 infections, highlighting the vulnerability of the aging immune system (Mueller et al. 2020; Nikolich‐Zugich et al. 2020). Recent studies of centenarians have provided valuable insights into the immune system's role in longevity. One such study indicated that centenarians often possess unique immune profiles contributing to their exceptional longevity, suggesting that immune function is a crucial determinant of lifespan (Karagiannis et al. 2023). This reinforces that understanding and enhancing immune function could promote healthy aging and longevity.

Our previous research has demonstrated the potential of Toll‐like receptor 5 (TLR5) as a novel geroprotector target. TLR5 is a pattern recognition receptor of the innate immune system that explicitly recognizes bacterial flagellin. Unlike other TLRs, which primarily induce pro‐inflammatory responses upon activation, TLR5 has been shown to play a multifaceted role in modulating immune responses, metabolism, and tissue regeneration. One significant example of TLR5's unique capabilities comes from studies on the mucosal delivery of flagellin‐containing fusion proteins. These studies have shown that such treatments improve the immune function of aged mice against pneumonia infection (Lim et al. 2015) and lead to systemic functional recovery across various aging tissues. Unlike traditional geroprotectors, which often focus on single pathways, TLR5 activation exerts broad‐spectrum effects, including enhancing brain metabolic functions, suppressing chronic inflammation, extending lifespan, and improving overall health (Lim et al. 2024). Furthermore, TLR5's metabolic and anti‐inflammatory protective functions have been reported, suggesting that TLR5 performs additional roles beyond inducing innate immunity.

Additionally, TLR5 has been reported to play a significant role in the liver. As an immune organ, the liver is continuously exposed to systemic or intestinal‐derived pathogens and plays an essential role in detecting and defending against pathogens (Racanelli and Rehermann 2006). Previous studies have shown that TLR5 functions in the liver to act as a vascular firewall, trapping and eliminating commensal bacteria entering the intestinal or systemic vasculature and suppressing hepatic inflammatory responses (Burdelya et al. 2013; Etienne‐Mesmin et al. 2016; Yang and Yan 2017). Activation of TLR5 in the liver prevents liver fibrosis and improves liver regeneration. According to these studies, the cause of liver disease in the liver‐specific TLR5 knockout is related to the increased expression of pro‐inflammatory cytokines, which are dependent on the Nod‐like receptor C4 inflammasome and are rescued by microbiota ablation (Etienne‐Mesmin et al. 2016). TLR5 signaling has emerged as a key player in promoting mouse liver regeneration, indicating its potential as a therapeutic target for liver repair. This notion gains further traction with the discovery of the synergistic effects between the TLR5 agonist CBLB502 and its downstream effector IL‐22 in mitigating liver injury (Melin et al. 2021; Zhang et al. 2021). These findings suggest a promising combination therapy bolsters liver protection and fosters tissue recovery. However, the underlying mechanisms involved in the functional role of TLR5 in liver diseases remain unclear.

To further understand the mechanism of aging function recovery mediated by TLR5, we monitored the changes in the TLR5 knockout (KO) mice with age. Interestingly, we found that metabolic abnormalities, particularly in liver function, were more pronounced than other age‐related changes, underscoring the significant role of liver metabolism in the aging process facilitated by TLR5. In this study, we aimed to identify the role of TLR5 in regulating immunometabolism during aging and to further verify its therapeutic effect in metabolic diseases. These findings suggest that TLR5 could be a valuable target for developing novel treatments that specifically address the metabolic challenges associated with aging.

## 2 Methods

### 2.1 Generation of Fc‐Flagellin

Fc‐flagellin was designed with hIgG4 Fc linked to the C‐terminus of flagellin derived from *Bacillus subtilis* using the GGGGS linker. An additional mutation (S228P) was introduced in the hinge region to optimize the protein further. The Fc‐fused *Bacillus subtilis* flagellin was expressed using Chinese Hamster Ovary (CHO) cells with the mammalian expression plasmid pFUSE‐hIgG4. After expression, the proteins were purified by Fc‐affinity and size exclusion chromatography. The purified product was stored at −80°C.

### 2.2 Animals and Experimental Protocols

Five‐week‐old male C57BL/6J mice were obtained from Orient Bio Inc. (Seoul, Korea). TLR5 knockout (KO) mice (backcrossed to a C57BL/6J background for ten generations) were purchased from The Jackson Laboratory (Bar Harbor, ME, USA). Mice were inbred by homozygotes mating.

All mouse procedures were conducted according to the Animal Care and Use Committee guidelines of Chonnam National University (approval number: CNU IACUC‐H‐2019‐23). All mice were kept in a specific pathogen‐free facility under controlled light (07:00 am–07:00 pm) and temperature (22°C ± 2°C) conditions. The mice were used for methionine–choline‐deficient (MCD) and amylin liver NASH (AMLN) diet‐induced liver disease models at 5 weeks old. After 1 week of adaptation, C57BL/6J mice with similar body weights were randomly assigned to each experimental group. Details about animal experiments are provided in the supplementary method.

### 2.3 Biochemical Analysis

Serum levels of alkaline phosphatase (ALP), aspartate aminotransferase (AST), alanine aminotransferase (ALT), triglyceride (TG), and total cholesterol (T.CHO) were determined using a blood chemistry analyzer (AU480, Beckman Coulter, Germany) according to the manufacturer's protocol. Hepatic triglyceride (TG) levels were measured using a kit (MAK266, Sigma‐Aldrich). Homogenization of liver tissue was performed in PBS containing 1% Triton X‐100 and centrifuged at 10,000 rpm for 5 min. The supernatants were used in the TG quantification assay according to the manufacturer's protocol.

### 2.4 Histological Analysis

Formalin‐fixed, paraffin‐embedded mouse liver sections were stained with hematoxylin and eosin (H&E) to evaluate steatosis and inflammatory cell infiltration. The non‐alcoholic fatty liver activity score (NAS) was assessed based on Kleiner's criteria by scoring steatosis (0–3), lobular inflammation (0–3), and hepatocyte ballooning (0–2) (Kleiner et al. 2005). Details about histological analysis are provided in the supplementary method.

### 2.5 Quantitative Real‐Time PCR

Total RNA was extracted using TRIzol Reagent (Thermo Fisher Scientific, Waltham, MA, USA) according to the manufacturer's instructions. After quantification using a Nanodrop, RNA was retrotranscribed into cDNA using an iScript cDNA synthesis kit (BioRad, Hercules, CA, USA). Quantitative Real‐Time PCR was performed using the Quant Studio 3 Flex System (Thermo Fisher Scientific). The relative RNA expression was normalized using the housekeeping gene GAPDH. Primers used for qRT‐PCR are listed in Table S1.

### 2.6 Western Blotting Analysis

Tissues and cells were homogenized in radioimmunoprecipitation assay (RIPA) buffer (50 mM Tris–HCl (pH 7.6), 150 mM NaCl, 5 mM ethylenediaminetetraacetic acid, 1% NP40, 0.1% sodium dodecyl sulfate, and protease inhibitors) and sonicated. Total protein was collected after centrifugation. Protein samples were separated using SDS‐PAGE and transferred onto polyvinylidene fluoride membranes. The membranes were incubated overnight with anti‐CD68 (NB600‐985; Novus Biologicals, Centennial, CO, USA) and anti‐GAPDH (SC‐365062; Santa Cruz Biotechnology) antibodies in a cold room. The membranes were then incubated with peroxidase‐conjugated anti‐rabbit and anti‐mouse secondary antibodies (Santa Cruz Biotechnology) for 1 h at RT and visualized using an enhanced chemiluminescence detection kit (Amersham ECL Kit; GE Healthcare, Buckinghamshire, UK). Protein expression was analyzed using ImageJ software.

### 2.7 Isolation of Primary Mouse Hepatic Stellate Cells and Hepatocytes

Primary mouse hepatic stellate cells (HSCs) and hepatocytes (PMHs) were isolated from the livers of WT and TLR5 KO mice by type IV collagenase (0.8 mg/ml) perfusion through the portal vein of mice anesthetized with isoflurane. Hepatocytes were filtered through a cell strainer (100 μm nylon, BD), washed with DMEM, resuspended in DMEM, centrifuged through 45% Percoll (P4937, Sigma‐Aldrich Inc.), and cultured in DMEM containing 10% fetal bovine serum (Kim et al. 2016). HSCs were isolated using the density gradient‐mediated separation method with the Nycodenz solution. The overlay cell Nycodenz solution was centrifuged at 1.380×*g* for 15 min, and the isolated cells from layer separation were cultured in DMEM media (Mederacke et al. 2015). To determine the purity of PMHs and HSCs, qRT‐PCR was performed using *Decornin* (*Dcn*) and *Albumin* (*Alb*), genes known as markers of HSCs and PMHs, respectively (Balaphas et al. 2022; Bartneck et al. 2015). Primers used for qRT‐PCR are listed in Table S1.

### 2.8 Metabolic Flux Analysis

The oxygen consumption rate (OCR) and extracellular acidification rate (ECAR) were measured using a Seahorse XF96 extracellular flux analyzer (Seahorse Bioscience, Billerica, MA, USA). Briefly, mouse HSCs were plated on Seahorse XF‐96‐well plates at a density of 2 × 10^5^ per well to achieve 80%–90% confluency during the assay. The plate was incubated overnight at 37°C to allow the cells to adhere. The following day, the medium was replaced with Seahorse XF medium, and the protocol was followed as described by the supplier of the mistress kit (Seahorse Bioscience). Basal levels of OCR and ECAR were recorded, followed by a mitochondrial stress test (1 μM oligomycin, 1 μM FCCP, 0.5 μM rotenone, and 0.5 μM antimycin A).

### 2.9 RNA‐Sequencing Analysis

Liver RNA was extracted using the Trizol/chloroform/isopropanol method (InvitrogenTM, Carlsbad, CA, USA) and sent to Ebiogen (Ebiogen Inc., Korea) for library preparation and sequencing. Library construction was performed using the QuantSeq 3′ mRNA‐Seq Library Prep Kit (Lexogen Inc., Austria) according to the manufacturer's instructions. In brief, each total RNA was prepared, an oligo‐dT primer containing an Illumina‐compatible sequence at its 5′ end was hybridized to the RNA, and reverse transcription was performed. After degradation of the RNA template, second strand synthesis was initiated by a random primer containing an Illumina‐compatible linker sequence at its 5′ end. The double‐stranded library was purified using magnetic beads to remove all reaction components. The library was amplified to add the complete adapter sequences required for cluster generation. The finished library is purified from PCR components. High‐throughput sequencing was performed as single‐end 75 sequencing using NextSeq 550 (Illumina Inc., USA). Sequencing data were deposited in the Gene Expression Omnibus (GEO) database of the National Center for Biotechnology Information (Edgar et al. 2002) with GEO Series accession number (GSE272622).

Annotation categories, including gene ontology of interest, were retrieved from QuickGO (https://www.ebi.ac.uk/QuickGO/annotations) and applied to the analysis workflow. Gene selection and graphical visualization were performed using the ExDEGA Graphic Plus software (Ebiogen Inc., Korea). Gene annotation enrichment analysis was conducted using the DAVID Functional Annotation Tool (https://david.ncifcrf.gov/summary.jsp) (Jiao et al. 2012), with a pathway enrichment analysis cutoff of fold change > 1.5 and *p* < 0.05. Hierarchical clustering analysis was performed with MeV 4.9.0 (Saeed et al. 2003) using Euclidean distance correlation as the distance metric and average linkage clustering (Eisen et al. 1998). The resulting clusters and heat maps were visualized with the same software. For senescence gene analysis, the SAUL_SEN_MAYO gene set was obtained from the GSEA database (https://www.gsea‐msigdb.org/gsea/index.jsp) and analyzed using ExDEGA and MeV 4.9.0 software with thresholds set at *p* < 0.05 and fold change > 1.5 (Saul et al. 2022).

### 2.10 Statistical Analysis

Statistical analysis was performed using Prism 9 software (GraphPad Inc., San Diego, CA, USA). Differences between the experimental groups were analyzed using one‐way or two‐way analysis of variance (ANOVA) with the Bonferroni post hoc test for single or multiple comparisons. The data are represented as mean ± SD and were considered statistically significant when *p* values were < 0.05. All experiments were performed at least three times.

## 3 Results

### 3.1 Age‐Dependent Metabolic Abnormalities in TLR5 Knockout Mice

Building on our previous observations of the multi‐functional improvement associated with TLR5 stimulation in aged mice (over 25 months), including metabolic benefits (Lim et al. 2024), we investigated the age‐ and gender‐related changes in TLR5 knockout (KO) mice to understand the underlying mechanisms.

**FIGURE 1:**
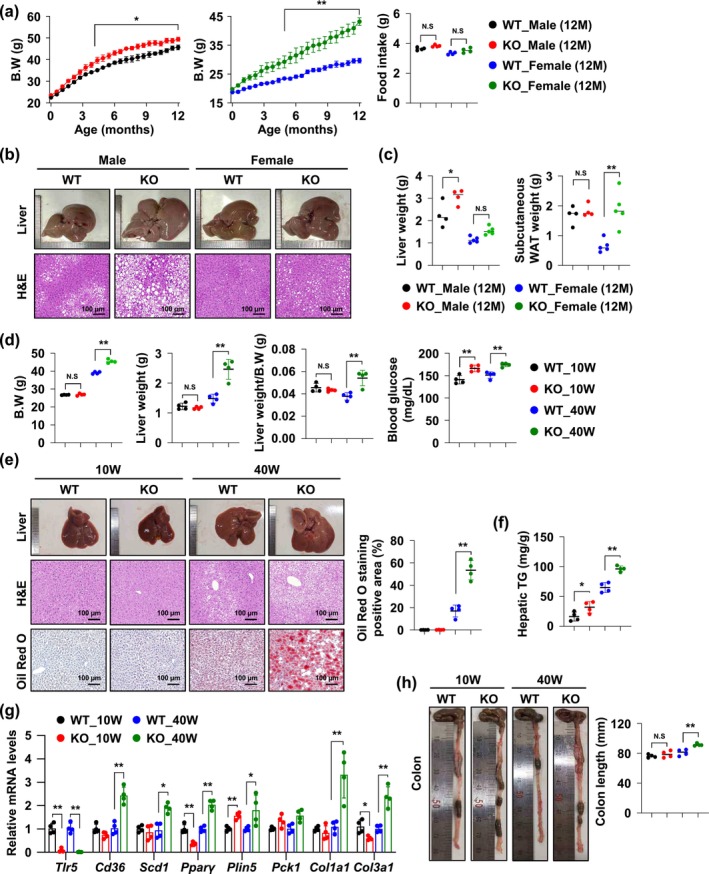
TLR5 deficiency causes fatty liver disease in mice depending on age. (a) Comparison of body weight and food intake between wild‐type (WT) and TLR5 knockout (KO) by week of age in male and female mice (*n* = 4). (b) Liver image and histology of WT and TLR5 KO from 12‐month‐old male and female mice. Liver sections were stained with Hematoxylin and Eosin (H&E). Scale bar, 100 μm. (c) Liver weight and subcutaneous WAT weight of WT and TLR5 KO from 12‐month‐old male and female mice. (d) Body weight (B.W.), liver weight, liver weight/B.W., and blood glucose level of 10‐ and 40‐week‐old WT and TLR5 KO mice. (e) Liver image and histology of 10‐ and 40‐week‐old WT and TLR5 KO mice. Liver sections were stained with H&E and Oil Red O. The result of the computer‐based analysis of the Oil Red O‐stained positive area is presented (right panel, *n* = 4 in each group). Scale bar, 100 μm. (f) Hepatic triglyceride (TG) levels in 10‐ and 40‐week‐old WT and TLR5 KO mice liver extracts. (g) The mRNA levels of the indicated genes in 10‐ and 40‐week‐old WT and TLR5 KO mice were measured by qRT‐PCR. Values for 10‐ and 40‐week‐old WT mice were set to 1 (*n* = 4). (h) Colon images and lengths (right panel) in 10‐ and 40‐week‐old WT and KO mice (*n* = 4). All values are presented as the mean ± SD. Statistical significance was measured using one‐way or two‐way ANOVA with the Bonferroni post‐test. *p < 0.05, **p < 0.005, N.S. statistically not significant.

As a result of observing the body weight of WT and TLR5 KO males and females until 52 weeks (12 months) of age, despite no difference in food intake, both males and females significantly increased in TLR5 KO mice compared to WT mice from 22 weeks of age (Figure 1a). At 12 months of age, TLR5 KO male mice showed, significantly increased liver weight and decreased gonadal white adipose tissue (WAT) compared to WT male mice. In TLR5 KO female mice, the weight of subcutaneous WAT, gonadal WAT, brown adipose tissue (BAT), and fasting blood glucose was significantly increased compared to WT female mice. There was no difference in colon length between WT and TLR5 KO mice in both males and females (Figure 1b,c and Figure S1). Histological analysis of hematoxylin–eosin (H&E) staining revealed that intracellular lipid deposition in the livers of 12‐month‐old male TLR5 KO exhibited a significant increase compared to that of male WT mice. In females, lipid deposition increased in the livers of TLR5 KO mice compared to WT mice, although not as much as in males (Figure 1c).

Next, to further monitor age‐related liver disease caused by TLR5 deficiency, we compared metabolic disorders between 10‐ and 40‐week‐old WT and TLR5 KO male mice. In 10‐week‐old WT and TLR5 KO mice, there were no differences in body weight, liver weight, or liver weight/body weight. However, in 40‐week‐old TLR5 KO mice, these weights were significantly increased compared to WT mice. Fasting glucose levels were significantly increased in TLR5 KO mice at 10 and 40 weeks compared to those in WT mice (Figure 1d). Histological analysis of H&E staining revealed hepatic steatosis in the liver tissues of 40‐week‐old TLR5 KO mice, which was not observed in the WT mice as much as in TLR5 KO mice. Oil‐Red O staining showed that intracellular lipid deposition in the livers of 40‐week‐old WT and TLR5 KO mice increased compared to 10‐week‐old mice. Moreover, the livers of 40‐week‐old TLR5 KO mice exhibited a significantly elevated level of intracellular lipid deposition compared to those of 40‐week‐old WT mice (Figure 1e). The increased hepatic triglyceride (TG) content further corroborated the visual evidence of lipid droplet enlargement within hepatocytes. The hepatic TG level of TLR5 KO mice was significantly increased compared to WT mice and further increased at 40 weeks of age (Figure 1f). The expression profiles of genes associated with lipid metabolism, fibrosis, and gluconeogenesis in liver tissues presented significant upregulation in TLR5 KO mice compared to WT mice, underscoring the metabolic impact of TLR5 deficiency (Figure 1g and Figure S1c).

However, despite considerable metabolic and hepatic alterations, no correlating inflammatory responses were observed in colonic tissues at 10 or 40 weeks (Figure 1h). These results suggest the predominant interpretation that TLR5‐related metabolic disorders are secondary to dysbiosis and gut inflammation. To evaluate the significance of TLR5 in intestinal inflammation, we utilized a well‐established model of chemically induced colitis. WT and TLR5 KO mice were administered dextran sulfate sodium (DSS) in their drinking water, a standard method for inducing inflammatory bowel disease (IBD) symptoms. As shown in Figure S2, both WT and TLR5 KO mice showed similar changes in body weight throughout DSS induction, with no significant differences between the groups. This phenotype continued across the 5‐day observation period, suggesting that TLR5 deletion did not alter the physical inflammatory responses to DSS‐induced stress.

Furthermore, the Disease Activity Index (DAI), which combines clinical indicators of IBD severity, including weight loss, stool consistency, and bleeding, followed the same pattern in WT and TLR5 KO mice treated with DSS. This result indicated that TLR5 deficiency does not exacerbate the clinical signs of DSS‐induced colitis. These findings were corroborated by visual and measured assessments of colon length and liver weight, demonstrating that TLR5 KO mice did not differ significantly from their WT counterparts regarding colonic shortening or liver‐to‐body weight ratio after DSS treatment.

These observations suggest that TLR5's role in gut immunity may not be as critical in the development of DSS‐induced colitis as previously thought, and TLR5 KO mice exhibit increased obesity and metabolic dysfunction with age, indicating that liver metabolic function abnormalities may be a more sensitive trigger than intestinal inflammation.

### 3.2 Enhanced Susceptibility to MCD Diet‐Induced Liver Pathology in TLR5 Knockout Mice: Implications for Metabolic Disease Protection

**FIGURE 2:**
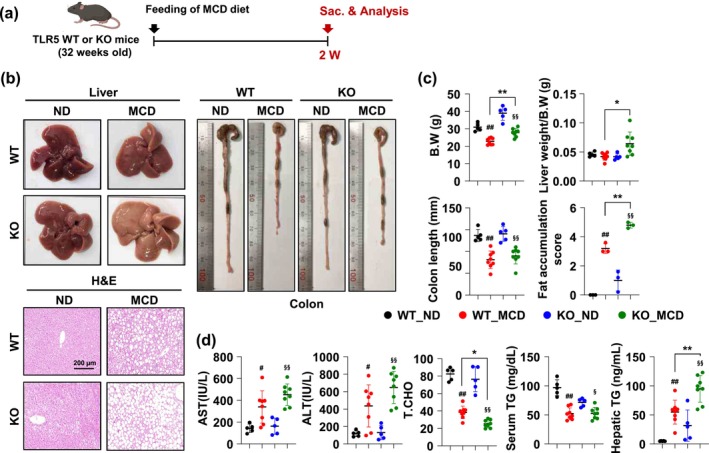
TLR5 deficiency exacerbates the risk for steatosis in MCD diet mice. (a) Experimental outline. (b) Liver and colon images of normal diet (ND) and methionine‐choline deficient (MCD) diet in WT and TLR5 KO mice. Liver sections were stained with H&E (bottom panel). (c) Body weight (B.W.), liver weight/B.W., and colon length were shown (*n* = 8 per group). Fat accumulation score was measured by the number and size of lipid droplets as well as the degree of steatosis (*n* = 3). (d) Levels of aspartate aminotransferase (AST), alanine aminotransferase (ALT), total cholesterol (T.CHO), and triglycerides (TG) in serum and liver tissue. All values are presented as the mean ± SD. Statistical significance was measured using two‐way ANOVA with the Bonferroni post‐test. *p < 0.05, **p < 0.005, ^#^ *p* < 0.05, ^##^ *p* < 0.005 compared with the normal diet WT group. ^§^ *p* < 0.05, ^§§^ *p* < 0.005 compared with the normal diet TLR5 KO group.

To assess the impact of TLR5 on liver metabolic disease resistance, we administered a normal diet (ND) and methionine–choline deficient (MCD) diet to WT and TLR5 KO mice, which are known to induce liver fibrosis (Figure 2a). Visual assessment and histological analysis revealed that TLR5 KO mice displayed more severe liver damage and fibrosis symptoms than their WT counterparts when subjected to the MCD diet. Histological staining with H&E revealed a significant disruption of the liver tissue architecture and higher fat accumulation scores in TLR5 KO mice on the MCD diet than in WT mice on the MCD diet (Figure 2b,c). Consistent with these data, biochemical analysis showed that the liver enzymes AST and ALT, which are biomarkers indicative of liver damage, were significantly increased in mice fed the MCD diet and were considerably higher in TLR5 KO mice than in WT mice (Figure 2d). Additionally, markers of inflammation and fibrosis in the liver (Galectin‐3 and CD68) were significantly increased in TLR5 KO mice compared to WT mice (Figure S3). This was further evidenced by elevated serum and liver triglyceride levels in TLR5 KO mice, which is consistent with the characteristic symptoms of metabolic disorders. These data suggest that TLR5 deficiency leads to an increased propensity for lipid deposition within the liver, thereby exacerbating the risk of steatosis.

### 3.3 TLR5 Deficiency Induces and Exacerbates Gene Expression Changes in Liver Tissue Similar to Those Caused by MCD Diet in Mice

**FIGURE 3:**
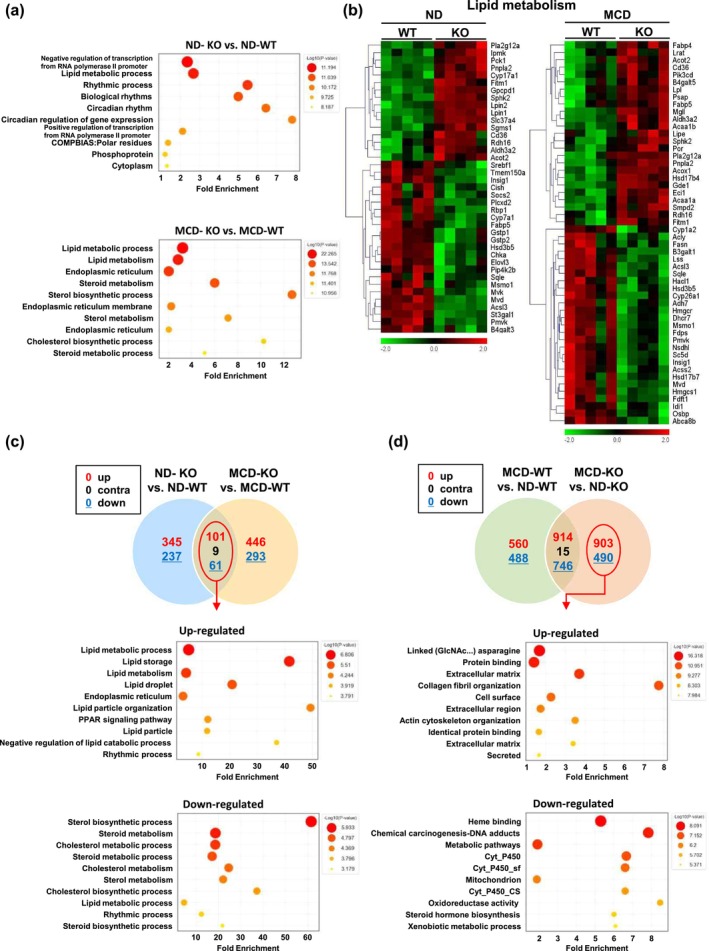
TLR5 deficiency regulates the expression of lipid metabolism‐related genes. 32‐week‐old wild‐type (WT) and TLR5 knockout (KO) mice were fed a normal diet (ND) and a methionine–choline‐deficient (MCD) diet for 2 weeks; the livers were collected, and RNA‐sequencing was performed. (a) Gene ontology (GO) analysis of ND‐TLR5 KO compared to ND‐WT mice (ND‐KO vs. ND‐WT, top) and MCD diet TLR5 KO compared to MCD diet WT mice (MCD‐KO vs. MCD‐WT, bottom). (b) Hierarchical clustering shows changes in gene expression in ND TLR5 KO mice compared with ND WT mice (left) and in MCD diet TLR5 KO mice compared with MCD diet WT mice (right). (c) Venn diagram of genes changed in ND‐KO vs. ND‐WT and MCD‐KO vs. MCD‐WT mice. GO analysis of genes showing up‐ and down‐regulated expression among overlapping genes (bottom). (d) Venn diagram of genes changed in MCD‐WT vs. ND‐WT and MCD‐KO vs. ND‐KO. GO analysis of genes showing up‐ and down‐regulated expression in the TLR5 KO group only (bottom).

Following our examination of diet‐induced liver pathology in WT and TLR5 KO mice, we conducted comprehensive RNA sequencing analysis to discern the molecular underpinnings of the observed phenotypic changes. The volcano plot shown in Figure S4a highlights the differential gene expression between ND and MCD diets in TLR5 KO mice compared to WT mice (*p* < 0.05, fold change [FC] > 1.5). Gene ontology (GO) analysis revealed that, compared to the WT group, ND resulted in altered lipid metabolism‐ and circadian rhythm‐related genes in mice with TLR5 deficiency. GO analysis of mice fed the MCD diet revealed a predominant effect on genes involved in lipid, steroid, and cholesterol biosynthesis (Figure 3a, Table S2). The representative heat map of gene expression in lipid metabolism, sterol metabolism, immune response, and rhythm processes revealed changes in expression observed in the WT and TLR5 KO groups under ND and MCD diet conditions. (Figure 3b and Figure S4b–d). Consistent with the RNA‐seq data, qRT‐PCR analysis revealed that the expression levels of lipid uptake gene (*Cd36*), lipid metabolic process genes (*Lpin1*, *Lpin2*, *Pck1*, *Apoa4*, and *Pparα*), fatty acid metabolism genes (*Acot1* and *Acot2*), and lipid droplet genes (*Plin5*, *Cidea*, and *Cidec*) were upregulated in the liver of ND‐TLR5 KO mice compared to those in ND‐WT mice, whereas the expression levels of lipid biosynthesis process genes (*Socs2*, *Insig1*, and *Srebp1*) were downregulated in the livers of ND‐TLR5 KO mice (Figure S4e). The expression levels of lipid and fatty acid uptake genes (*Cd36* and *Fabp4*), lipid catabolic process and fatty acid β‐oxidation genes (*Acox1, Acot1, Acot2, Eci1*, and *Acaa1b*), and lipid droplet genes (*Cidea* and *Cidec*) were upregulated in the liver of MCD‐TLR5 KO mice compared to those in MCD‐WT mice, whereas the expression levels of lipid biosynthesis process genes (*Fas, Insig1*, and *Hmgcr*) were downregulated in the livers of MCD‐TLR5 KO mice (Figure S4f).

In addition, TLR5 KO on ND and MCD diets (*p* < 0.05, [FC] > 1.5) altered the expression of 753 and 910 genes, respectively, and 171 genes were altered under both conditions. TLR5 KO in ND and MCD diets resulted in the up‐regulation of genes involved in lipid metabolism, lipid storage, and lipid droplets. Conversely, genes associated with sterol and cholesterol metabolism were downregulated. Genes related to rhythmic processes were both upregulated and downregulated (Figure 3c). GO analysis was performed on differentially expressed genes (DEGs), excluding overlapping genes, in TLR5 KO mice fed an ND or MCD diet compared to WT mice (Figure S4g). In comparison to TLR5 KO and WT mice, transcription‐related genes exhibited a significantly altered expression profile in ND. In contrast, genes related to immune response and sterol metabolism exhibited considerably altered expression profiles in the MCD diet. Next, we performed an analysis to exclude overlapping genes in response to the MCD diet in WT and TLR5 KO mice and to determine gene changes only in TLR5 KO mice. The study revealed that genes exhibiting alterations in expression only in TLR5 KO mice on the MCD diet included 903 upregulated and 490 downregulated genes. GO analysis revealed that genes involved in fibrosis‐related extracellular matrix, collagen fibril organization, and actin cytoskeleton organization were upregulated in TLR5 KO mice fed an MCD diet (Figure 3d).

Interestingly, when comparing the TLR5 deficiency model with the MCD diet WT model, we found that TLR5 deficiency alone mirrored the gene changes induced by the MCD diet. GO analysis revealed that the gene expression profiles of TLR5 KO mice fed the ND and WT mice fed the MCD diet were remarkably similar (Figure S5). The expression patterns of specific genes involved in lipid metabolism and circadian regulation show similar changes between the two groups. Notably, circadian‐related genes exhibited changes in response to the MCD diet and absence of TLR5. The modifications in circadian‐related genes observed under these two conditions also exhibited comparable KEGG pathways. The mRNA level of *Nr1d1* (*Rev‐Erbα*), which plays a crucial role in regulating circadian rhythms and lipid metabolism, was increased in both WT mice on the MCD diet and TLR5 KO mice compared to WT mice on ND (Figure S5). In our previous study (Lim et al. 2024), we demonstrated that TLR5 plays a critical role in modulating aging and promoting longevity. Based on these findings, we analyzed genes related to senescence using the SenMayo gene set in the current study (Saul et al. 2022) to further investigate the relationship between TLR5 and cellular senescence. We observed that SenMayo genes, associated with cellular senescence, were significantly upregulated in TLR5 KO mice compared to WT mice under both ND and MCD diets. The significant upregulation of senescence‐related genes, including *Igfbp3, Angptl4, Tnfrsf1b, Gdf15*, and *p21*, in TLR5‐deficient mice under the MCD diet suggests that TLR5 deficiency may exacerbate cellular senescence under metabolic stress (Figure S6).

These results suggest that TLR5 plays a critical role in regulating various aspects of lipid metabolism, including uptake, catabolism, biosynthesis, storage, circadian rhythmic processes, and cellular senescence. TLR5 deficiency in the MCD diet also aggravated liver fibrosis by upregulating fibrosis‐related genes. These results suggest that TLR5 could be a key target for modulating lipid metabolism in liver metabolic diseases.

### 3.4 Age‐Related Effects of TLR5 Deficiency on Liver Metabolism by Primary Hepatocytes

**FIGURE 4:**
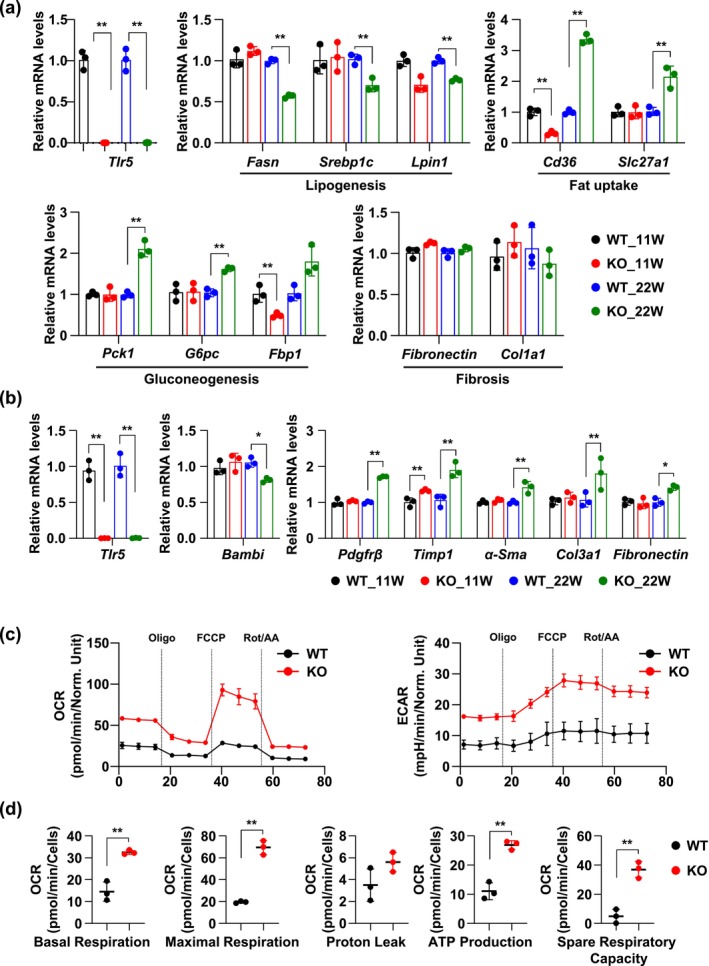
Age‐related effects of TLR5 deficiency in primary mouse hepatocytes and hepatic stellate cells. (a) The mRNA levels of the indicated genes were measured by qRT‐PCR for primary mouse hepatocytes (PMHs) of 11 and 22‐week wild‐type (WT) and TLR5 knockout (KO) mice. Values for 11‐ and 22‐week WT mice were set to 1 (*n* = 3). (b) The mRNA levels of the indicated genes were measured by qRT‐PCR for primary mouse hepatic stellate cells (HSCs) of 11 and 22‐week WT and TLR5 KO. Values for 11‐ and 22‐week WT mice were set to 1 (*n* = 3). (c and d) XF Cell Mito Stress test and respiratory parameters of freshly isolated HSCs from 22 weeks WT and TLR5 KO mice. The responses were to 1 μM oligomycin (Oligo), 1 μM FCCP (carbonyl cyanide 4‐(trifluoromethoxy) phenylhydrazone), or 0.5 μM Rot/AA (rotenone/antimycin A) in glucose‐based media (*n* = 3 wells per group). Data are representative of at least two independent experiments. All values are presented as the mean ± SD. Statistical significance was measured using two‐way ANOVA with the Bonferroni post‐test (a and b) and Student's *t* test (d). *p < 0.05, **p < 0.005.

To further investigate the functional role of TLR5 in hepatic lipid metabolism, primary mouse hepatocytes (PMHs) were isolated from WT and TLR5 KO mice at an age when body weight differences and TLR5 deficiency‐related changes became apparent. The purity of the isolated cells was confirmed by qRT‐PCR using *Decorin* (*Dcn*) and *Albumin* (*Alb*), markers of HSCs and PMHs, respectively (Figure S7) (Balaphas et al. 2022; Bartneck et al. 2015). At 11 weeks, hepatocytes from WT and TLR5 KO mice exhibited no significant differences in the expression of genes involved in lipogenesis, fat uptake, gluconeogenesis, and fibrosis (Figure 4a). This result suggests that TLR5 deficiency does not markedly affect hepatic metabolic pathways at a younger age within 11 weeks. However, after 22 weeks, notable changes were apparent in the PMH from TLR5 KO mice. The expression levels of several key genes related to lipogenesis (*Fasn*, *Srebp‐1c*, and *Lpin1*), fat uptake (*Cd36* and *Slc27a1*), and gluconeogenesis (*Pck1*, *G6pc*, and *Fbp1*) were significantly altered in TLR5 KO hepatocytes. However, fibrosis‐related genes (*Fibronectin* and *Col1a1*) showed no significant changes (Figure 4a). These data indicated that the TLR5 KO group exhibited alterations in metabolic disease genes with age, suggesting the potential for age‐related changes in liver metabolism resulting from TLR5 deficiency.

### 3.5 Age‐Related Effects of TLR5 Deficiency on Liver Fibrosis by Primary Hepatic Stellate Cells

Hepatic stellate cells (HSCs) play an essential role in the fibrosis process and are the leading cause of collagen expression and deposition by transdifferentiating from a quiescent to an activated state (Tsuchida and Friedman 2017; Zhang et al. 2016). To determine the TLR5‐dependent effect in HSCs, mouse primary HSCs were isolated from WT and TLR5 KO mice. We specifically chose to use 22‐week‐old mouse HSCs, since primary cell isolation from aging livers often yields insufficient cell numbers, increased risk of contamination, and challenges of large‐scale analyses (Mederacke et al. 2015). By using 22‐week‐old mice, we were able to isolate sufficient quantities of viable HSCs for comprehensive gene expression and oxygen consumption rate (OCR) measurements. The absence of TLR5 at 22 weeks led to a substantial increase in the activation state and fibrosis marker genes (*Pdgfrβ, Timp1, α‐Sma, Col3a1*, and *Fibronectin*) with repression of the quiescent state marker (*Bambi*) (Figure 4b), pointing to TLR5's role in modulating fibrosis in hepatic cells. Furthermore, metabolic assessment through measurements of oxygen consumption rates (OCRs) in Figure 4c,d reflects an activation in the mitochondrial function of TLR5 KO cells, with increased basal respiration, maximal respiration, and spare respiratory capacity, indicating the activation state of HSC for progressing fibrosis. This suggests that TLR5 may be integral to maintaining mitochondrial efficiency and overall cellular metabolism.

Together, these findings highlight the age‐dependent roles of TLR5 in liver metabolism, proposing that TLR5 deficiency has a more pronounced impact on the progression of metabolic dysregulation and fibrosis in hepatic cells, which could be vital in understanding the pathogenesis of liver diseases.

### 3.6 Evaluating the Therapeutic Efficacy of TLR5 Stimulation in Fatty Liver Disease Models

**FIGURE 5:**
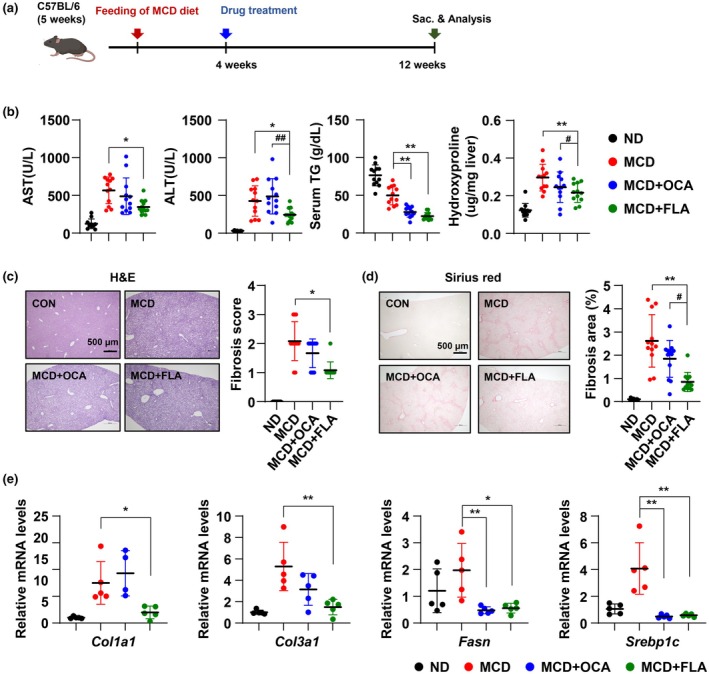
TLR5 stimulation mitigates liver damage and fibrosis in the MCD diet‐induced mice model. Wild‐type (WT) mice were fed a methionine–choline deficient (MCD) diet for 12 weeks. 4 weeks after starting the MCD diet, WT mice were administered with OCA (Obeticholic acid, 10 mg/kg), TLR5 ligand (FLA, Flagellin), or vehicle by oral gavage (once daily) and intravenous injection (twice weekly), respectively. Serum and liver samples were collected as shown in the experimental outline (a). (b) Serum alanine aminotransferase (ALT), aspartate aminotransferase (AST), triglycerides (TG), and hydroxyproline levels were determined (*n* = 12 in each group). (c and d) Representative images of the liver section stained with Hematoxylin and Eosin (H&E) and Sirius red to examine histology (scale bar 500 μm). Computer‐based morphometric analysis is shown (right graph, *n* = 12 in each group). (e) The mRNA levels of indicated genes were measured by qRT‐PCR. The values for the ND group were set to 1. All values are presented as the mean ± SD. Statistical significance was measured using two‐way ANOVA with the Bonferroni post‐test. *p < 0.05, **p < 0.005, ^#^ *p* < 0.05, ^##^ *p* < 0.005 compared to the MCD diet in the OCA treatment group.

To investigate whether TLR5 stimulation could improve liver metabolic disorders, we studied the therapeutic effects in the methionine‐choline deficient (MCD) diet‐induced fatty liver disease model. We administered a TLR5 ligand (flagellin, FLA) and compared its effectiveness with that of obeticholic acid (OCA), a known therapeutic candidate for liver diseases (Figure 5a). The group treated with the TLR5 ligand showed lower AST and ALT levels than the OCA‐treated group. Serum triglyceride and hydroxyproline levels were lower in mice treated with FLA than in those treated with OCA (Figure 5b). Histological assessments, including H&E staining and Sirius Red staining for fibrosis, provided further insights. While all mice on the MCD diet showed some liver damage and fibrosis, mice receiving the TLR5 ligand demonstrated less tissue damage and fibrosis. Lower fibrosis scores and reduced fibrotic area reflect these findings (Figure 5c,d). The fibrosis (*Col1a1* and *Col3a1*) and lipogenesis (*Fasn* and *Srebp1c*) related genes that were increased by the MCD diet were decreased by TLR5 ligand treatment (Figure 5e).

**FIGURE 6:**
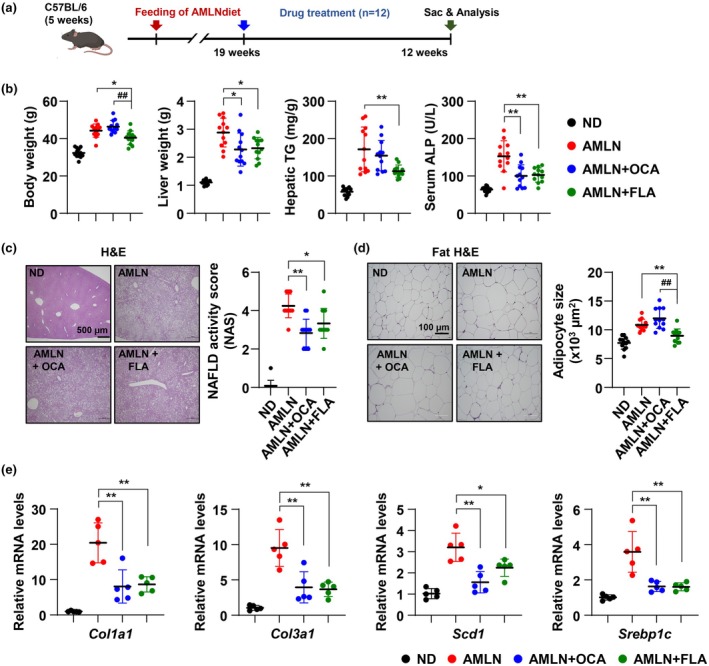
TLR5 stimulation mitigates fatty liver disease in the AMLN diet‐induced mice model. WT mice were fed an amylin liver NASH (AMLN) diet for 31 weeks. Nineteen weeks after starting the AMLN diet, WT mice were administered with OCA (Obeticholic acid, 30 mg/kg), TLR5 ligand (FLA, Flagellin), or vehicle by oral gavage (once daily) and intravenous injection (once weekly), respectively. (a) Experimental outline. (b) Body weight, liver weight, hepatic triglycerides (TG) level, and serum alkaline phosphatase (ALP) level were determined (*n* = 12 in each group). (c and d) Representative images of the liver (c) and epididymal fat (d) section stained with Hematoxylin and Eosin (H&E) to examine histology (scale bar 500 and 100 μm, respectively). NAFLD activity score (NAS) and computer‐based adipocyte size analysis were shown (right graph, *n* = 12 in each group). (e) The mRNA levels of indicated genes were measured by qRT‐PCR. The values for the ND group are set to 1. All values were presented as the mean ± SD. Statistical significance was measured using two‐way ANOVA with the Bonferroni post‐test. *p < 0.05, **p < 0.005. ^##^ *p* < 0.005 compared with the AMLN diet with the OCA treatment group.

Building upon our investigation of the therapeutic potential of TLR5 stimulation, we turned our attention to an advanced model of metabolic liver disease induced by the amylin liver NASH (AMLN) diet. The second model aimed to simulate the effects observed in non‐alcoholic steatohepatitis (NASH), a condition often characterized by significant lipid accumulation, inflammation, and fibrosis within the liver. After 19 weeks of feeding with the AMLN diet to induce a disease state akin to NASH, the mice received treatment interventions. The efficacy of a TLR5 ligand (FLA) or OCA was assessed against this backdrop and compared to the normal diet (ND) control and AMLN diet group (Figure 6a). The AMLN diet led to increased body weight, liver weight, hepatic triglycerides, and serum alkaline phosphatase (ALP) levels, indicating the diet's impact on inducing the hallmarks of NASH. However, when treated with FLA and OCA, these parameters were notably improved, and the TLR5 ligand demonstrated a potential therapeutic effect (Figure 6b). Histological evaluation supported these biochemical data. H&E staining revealed structural changes in the liver induced by the AMLN diet and subsequent modifications after treatment. Liver sections from OCA‐ and FLA‐treated mice showed reduced disease activity as measured by the NAFLD Activity Score (NAS), suggesting an amelioration of liver damage. In addition, the AMLN‐diet group showed increased adipocyte size, a hallmark of obesity and metabolic syndrome. In contrast, the FLA‐treated group exhibited a decrease in adipocyte size (Figure 6c,d). Fibrosis (*Col1a1* and *Col3a1*) and lipogenesis (*Fasn* and *Srebp1c*) related genes that were increased by the AMLN diet were significantly decreased by OCA and FLA treatment (Figure 6e). These findings suggest that TLR5 stimulation may be a promising avenue for the treatment of hepatic fibrosis and metabolic disorders associated with fatty liver disease. The comparable efficacy of the TLR5 ligand to OCA suggests that the role of TLR5 in liver health warrants further investigation, potentially positioning TLR5 ligands as novel therapeutics for liver disease treatment.

## 4 Discussion

In the discussion, we synthesized the complex interplay of TLR5's role in metabolic regulation and disease.

While most TLRs are known to mediate inflammation, contributing to the onset and progression of metabolic and various other diseases, TLR5 has received attention for its protective role in disease and its involvement in anti‐inflammatory processes and tissue regeneration. Unlike TLR4, which is typically associated with pro‐inflammatory responses and has been implicated in worsening metabolic syndrome and liver disease through inflammatory pathways (Kiziltas 2016), TLR5 appears to play a protective role. For instance, TLR5 activation not only helps in modulating the gut microbiota, reducing inflammation linked to metabolic disorders, but also enhances the production of interleukin‐10 (IL‐10), a potent anti‐inflammatory cytokine (Feng et al. 2023; Valentini et al. 2014; Vicente‐Suarez et al. 2007). TLR5 activation also induces the secretion of interleukin‐1 (IL‐1) receptor antagonists, reducing inflammasome damage. This critical finding highlighted TLR5's role in mitigating inflammatory responses within the gut–liver axis (Carvalho et al. 2011; Etienne‐Mesmin et al. 2016). Our findings suggest that, unlike other TLRs for which antagonists are sought for therapeutic development, TLR5 activation might protect against liver diseases, signifying a paradigm shift in the treatment approach for such conditions.

Amidst the evident age‐related metabolic perturbations, it is noteworthy that anticipated intestinal inflammation, commonly speculated to be a driver of metabolic syndrome through dysbiosis, was not prominently featured in our findings. The length of the colon, often reduced in inflammatory states, did not significantly differ between the TLR5 KO and WT mice with age. Moreover, in the induced IBD model in KO mice, no substantial differences emerged between the groups, suggesting that the metabolic irregularities observed may be attributed to intrinsic hepatic dysfunction rather than secondary to intestinal inflammation. This aspect of our results indicates the potential for TLR5's influence to be predominantly hepatic, warranting further exploration into its direct roles within the liver over secondary effects originating from gut inflammation. Recent TLR5 KO model research papers have also reported inconsistent results regarding the relationship between intestinal inflammation and metabolic abnormalities, suggesting that inflammation in the intestine may be a concurrent phenomenon rather than a first one. In addition, these studies did not observe the development of gut inflammation or colitis in mice with TLR5 deficiency, which suggests that the gut microbiota composition may be influenced by the animal‐rearing environment rather than a lack of TLR5 genes themselves (Carvalho et al. 2012; Leifer et al. 2014; Ubeda et al. 2012). In this study, we identified abnormalities in liver metabolism that are associated with aging in TLR5‐deficient mice.

Furthermore, investigations into the functional contribution of TLR5 within hepatocytes and hepatic stellate cells have revealed its crucial role in central hepatic functions. Although hepatic stellate cells exist in 5%–8% of the liver, their activation of hepatic stellate cells plays a vital role in liver fibrosis (Higashi et al. 2017). We observed that hepatic stellate cells are activated with aging when TLR5 is deficient. This phenomenon suggests that damage to TLR5‐deficient livers may exacerbate liver fibrosis. A comparison of gene analysis between TLR5 KO mice and the MCD diet showed that lipid metabolism and circadian rhythm exhibited very similar patterns. These findings suggest that TLR5 deficiency alone can induce metabolic and circadian rhythm changes similar to those caused by the MCD diet (Saer et al. 2024; Saran et al. 2020). This implies that TLR5 plays a crucial role in innate immunity and acts as an upstream regulator in various signaling pathways, significantly contributing to maintaining metabolic homeostasis. Previous studies have also suggested a connection between TLRs and circadian rhythms, particularly responses to commensal bacteria in intestinal epithelial cells (Papatriantafyllou 2013; Silver et al. 2012). These studies have demonstrated that TLRs are under circadian control and influence metabolic homeostasis. The literature increasingly indicates a close interplay between lipids and the circadian clock (Adamovich et al. 2015). Additionally, studies have examined the effects of drugs and the influence of susceptibility to infections involved in immune responses, such as inflammation, on the circadian clock (March et al. 2024; Ruan et al. 2021). Although further studies are needed to elucidate the relationship between TLR5 and the circadian clock, our findings extend these relationships to the liver, suggesting that TLR5 deficiency can disrupt circadian regulation and lipid metabolism, leading to metabolic disorders.

Furthermore, we investigated the therapeutic effects of TLR5 ligands in the MCD and AMLN models. Our results demonstrated that TLR5 activation can ameliorate liver metabolic diseases, indicating its potential as a drug target. This is particularly noteworthy as it contrasts with traditional approaches using TLR antagonists, showing that TLR5 agonists can effectively modulate liver metabolism and circadian rhythms. These findings suggest that targeting TLR5 with specific agonists could offer a novel therapeutic strategy for managing liver metabolic disorders, highlighting the importance of further research in this area. Although previous studies have reported that stimulating TLR5 can regulate various types of liver fibrosis, most of these studies focused on prevention models with simultaneous administration (Etienne‐Mesmin et al. 2016; Zhou et al. 2020). In contrast, our research expands the understanding of TLR5's effects by confirming its therapeutic efficacy after disease induction, thereby highlighting the broader potential of TLR5 as a therapeutic target.

Another critical point is that the observed age‐dependent metabolic abnormalities in TLR5 knockout mice, characterized by increased weight and liver steatosis, underscore the importance of TLR5 function in aging. These changes, absent in younger mice but manifesting as they age, highlight the potential of TLR5 to serve as a guardian against age‐associated metabolic decline. Although further studies on aging using TLR5 KO will be needed, this study demonstrated that senescence markers, such as *p21, Igfbp3, Angptl4*, and *Tnfrsf1β*, were significantly upregulated in TLR5 KO mice compared to WT mice on both ND and MCD diets. This suggests that TLR5 deficiency accelerates cellular senescence, which may contribute to the observed metabolic disturbances. This result is strongly related to our previously reported findings regarding the anti‐aging effects of nasal TLR5 stimulation (Lim et al. 2024). Integrating these prior findings, the age‐dependent abnormalities observed in TLR5 KO models in the current study have added significance. The progressive metabolic disturbances seen as mice age, specifically increased body weight and liver steatosis, are now viewed through a broader prism of TLR5's systemic role in aging. This context provides a more comprehensive understanding of age‐related metabolic changes and positions TLR5 as a pivotal factor in metabolic regulation and aging.

This study demonstrates that TLR5 deficiency in aged mice leads to metabolic problems, including obesity and liver fibrosis, primarily due to enhanced metabolic and fibrotic activities in hepatocytes and stellate cells. Importantly, these changes occur without colonic inflammation, highlighting the central role of TLR5 in liver metabolism. Furthermore, TLR5 stimulation with flagellin significantly improves liver health and metabolic functions, positioning TLR5 as a promising therapeutic target for preventing liver fibrosis and age‐related metabolic disorders. These findings underscore the importance of TLR5 in maintaining metabolic homeostasis and offer new insights into its potential as a therapeutic target for age‐associated diseases. Future research should focus on elucidating the detailed mechanisms linking TLR5, cellular senescence, and metabolic regulation to develop targeted therapies.

## Author Contributions

D.H.K. and K.A.C. designed the study. D.H.K., H.S.G., T.Q.T.N., and M.P. performed the experiments. E.J.J., D.Y.K., K.Y.K., and H.J.E. produced protein materials. D.H.K., H.P., and S.Y.K. analyzed the RNA‐Seq data. D.H.K., S.C.P., and K.A.C. analyzed the data. D.H.K. and K.A.C. wrote the manuscript. All authors have the opportunity to discuss the results and comments on the manuscript.

## Conflicts of Interest

The authors declare no conflicts of interest.

## Supporting information


Data S1.

Figures S1‐S7.

Table S1.

**Table S2.** Gene information from GO analysis.

## Data Availability

Data are available on request from the authors.
