# Supplementary material for: The Impact of Toll‐Like Receptor 5 on Liver Function in Age‐Related Metabolic Disorders
Source: Aging Cell. 2025 Feb 17;24(6):e70009. doi: 10.1111/acel.70009 (PMC12151890; doi:10.1111/acel.70009)
Supplement: Supplementary file 1 — Data S1. [file ACEL-24-e70009-s002.docx]

**The Impact of Toll-like Receptor 5 on Liver Function in Age-Related Metabolic Disorders**

**Methods**

**Animals and experimental protocols**

To compare study of age-dependent wild-type (WT) and TLR5 KO (n = 4 in each group) mice, we used 10- and 40-week-old mice. After fasting for six hours, mice were sacrificed. Serum, liver, and colon tissue samples were collected from mice.

To prepare the RNA-sequencing sample, age-matched (32 weeks old) wild-type (WT) and TLR5 KO mice were fed an MCD diet (A02082002B, RESEARCH DIETS Inc., New Brunswick, USA) and their corresponding regular diet (A02082003B, RESEARCH DIETS Inc., New Brunswick, USA) for two weeks. To study liver fibrosis, the MCD diet is typically administered for a period exceeding 4 weeks. However, to examine the RNA-seq analysis, we used the MCD 2-week diet model, which is the midpoint before fibrosis completely progresses. Animals were euthanized via intraperitoneal injection of pentobarbital sodium (200 mg/kg) at the end of the experiment. Blood samples were collected from the femoral artery for biochemical analysis. Livers were weighed and fixed in 10% formalin for histological analysis or snap‑frozen in liquid nitrogen, then stored at ‑80˚C in a freezer until required.

In the MCD experiment, the mice were initially divided into two groups. The first group (normal diet, ND group, n = 12) received a normal diet for four weeks, while the second group was fed an MCD (A02082002B, RESEARCH DIETS Inc., New Brunswick, USA). After four weeks, the mice on the MCD diet were randomly regrouped into three new subgroups: MCD diet group (n = 12), MCD + OCA group (n = 12), and MCD + FLA group (n = 12). The MCD + OCA group mice were administered obeticholic acid (OCA) daily at 10 mg/kg, dissolved in a 0.5% methylcellulose solution, for eight weeks. In contrast, the mice in the MCD + FLA group were administered Fc-flagellin (10 µg/head, weekly) for eight weeks.

In the AMLN experiment, the mice were randomly divided into two groups. The mice in the first group were given a control diet for 18 weeks (ND group, *n* = 12), while the mice in the other group were given an AMLN diet (D09100310, RESEARCH DIETS Inc., New Brunswick, USA). At week 18, the mice that were fed the AMLN diet were randomly regrouped into three new subgroups: AMLN diet group (*n* = 12), AMLN + OCA group (*n* = 12) and AMLN + FLA group (*n* = 12). Subsequently, mice in the AMLN + OCA group were orally administered with OCA (Sunshine Chemical, Wuhan, China) at a daily dose of 30 mg/kg, dissolved in a 0.5% methylcellulose solution, for 12 weeks. Mice in the AMLN + FLA groups received intravenous administrations of Fc-flagellin (10 µg/head, twice a week) for the same duration.

**Histological analysis**

H&E staining of epididymal adipose tissue samples was performed to measure mean adipocyte size. The stained sections were analyzed using ImageJ software (National Institutes of Health). Liver fibrosis was assessed by Sirius Red staining of formalin-fixed, paraffin-embedded mouse liver sections. Fibrosis was scored as F0 (no fibrosis), F1a (mild, zone 3, perisinusoidal fibrosis), F1b (moderate, zone 3, perisinusoidal fibrosis), F1c (portal/periportal fibrosis), F2 (perisinusoidal and portal/periportal fibrosis), F3 (bridging fibrosis) and F4 (cirrhosis). Quantitative analysis of fibrosis was performed by morphometry from digitalized Sirius Red-stained sections using the Aperio system after tuning the threshold of fibrosis detection under visual control. For ease of use, F1a, F1b, and F1c fibrosis were scored as 1. Immunohistochemical staining was performed using a Mouse on Mouse^TM^ basic kit (BMK-6100, Vector Laboratories, Burlingame, CA, USA) and a Vectastain^TM^ Elite ABC kit (PK-6102, Vector Laboratories). Liver sections were incubated with an anti-galectin three primary antibody (1:200 dilution, ab53082, Abcam, UK) and a biotinylated anti-mouse IgG secondary antibody (Vector Laboratories). Liver tissue was frozen in OCT compound, sectioned, and stained with Oil Red O. Stained sections were visualized using ImmPACT^TM^ DAB (SK-4105, Vector Laboratories) and then counterstained with Mayer's hematoxylin. Photomicrographs of the tissue sections were obtaine using an Olympus BX51 light microscope (LEICA, Wetzlar, Germany).
