# Supplementary material for: The Impact of Toll‐Like Receptor 5 on Liver Function in Age‐Related Metabolic Disorders
Source: Aging Cell. 2025 Feb 17;24(6):e70009. doi: 10.1111/acel.70009 (PMC12151890; doi:10.1111/acel.70009)
Supplement: Supplementary file 2 — Figures S1‐S7. [file ACEL-24-e70009-s001.pdf]

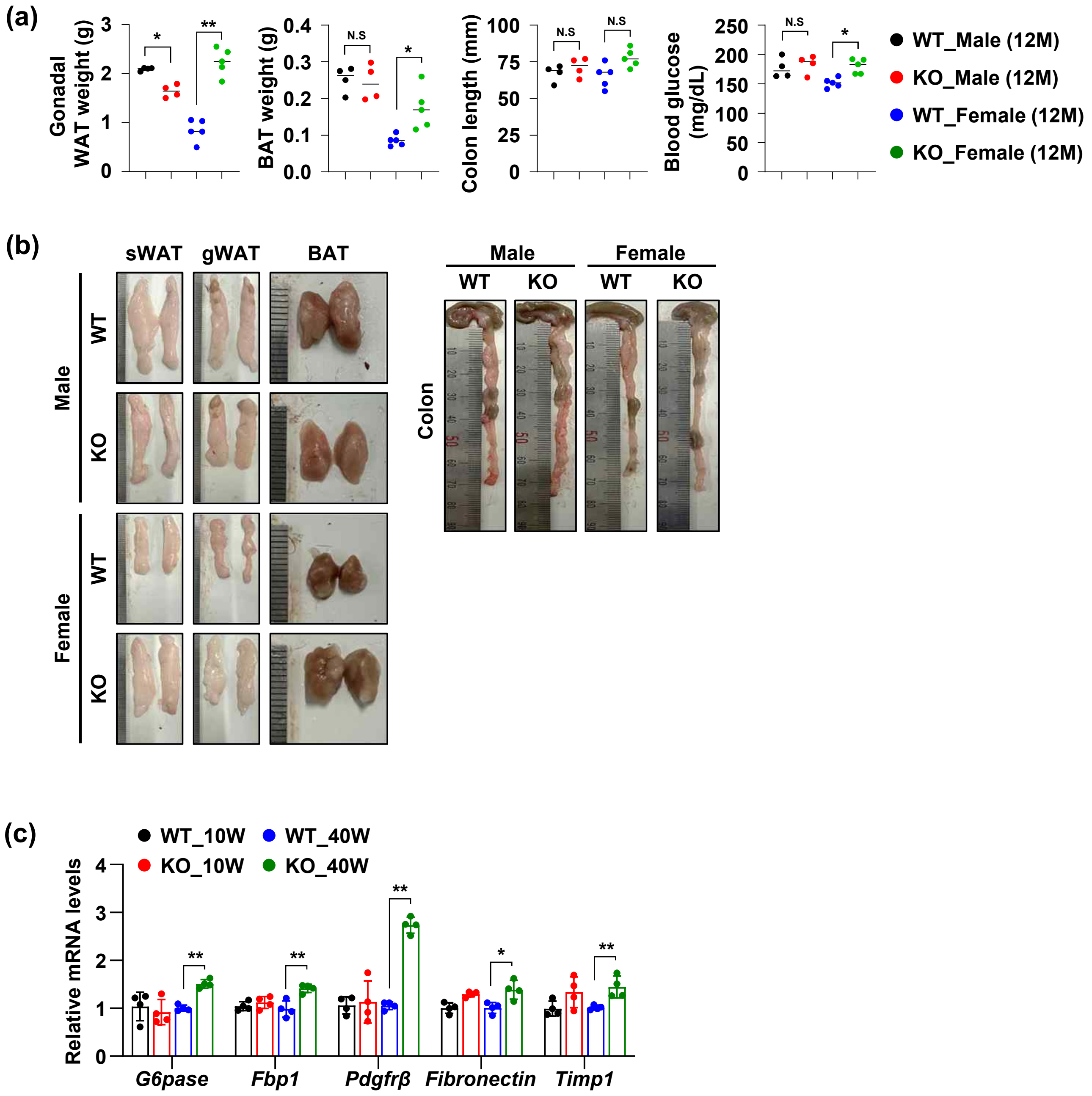

Figure S1. Effects of TLR5 deficiency on tissues in aged mice.

(a) Gonadal WAT weight, BAT weight, colon length, and WT and TLR5 KO blood glucose levels from 12-month-old male and female mice. (b) Tissue images of WT and TLR5 KO from 12-month-old male and female mice. (c) The mRNA levels of the indicated genes were measured by qRT-PCR. Values for 10- and 40-week-old WT mice were set to 1 (n = 4). All values are presented as the mean ± SD. Statistical significance was measured using one-way or two-way ANOVA with the Bonferroni post-test. *P < 0.05, **P < 0.005, N.S statistically not significant.

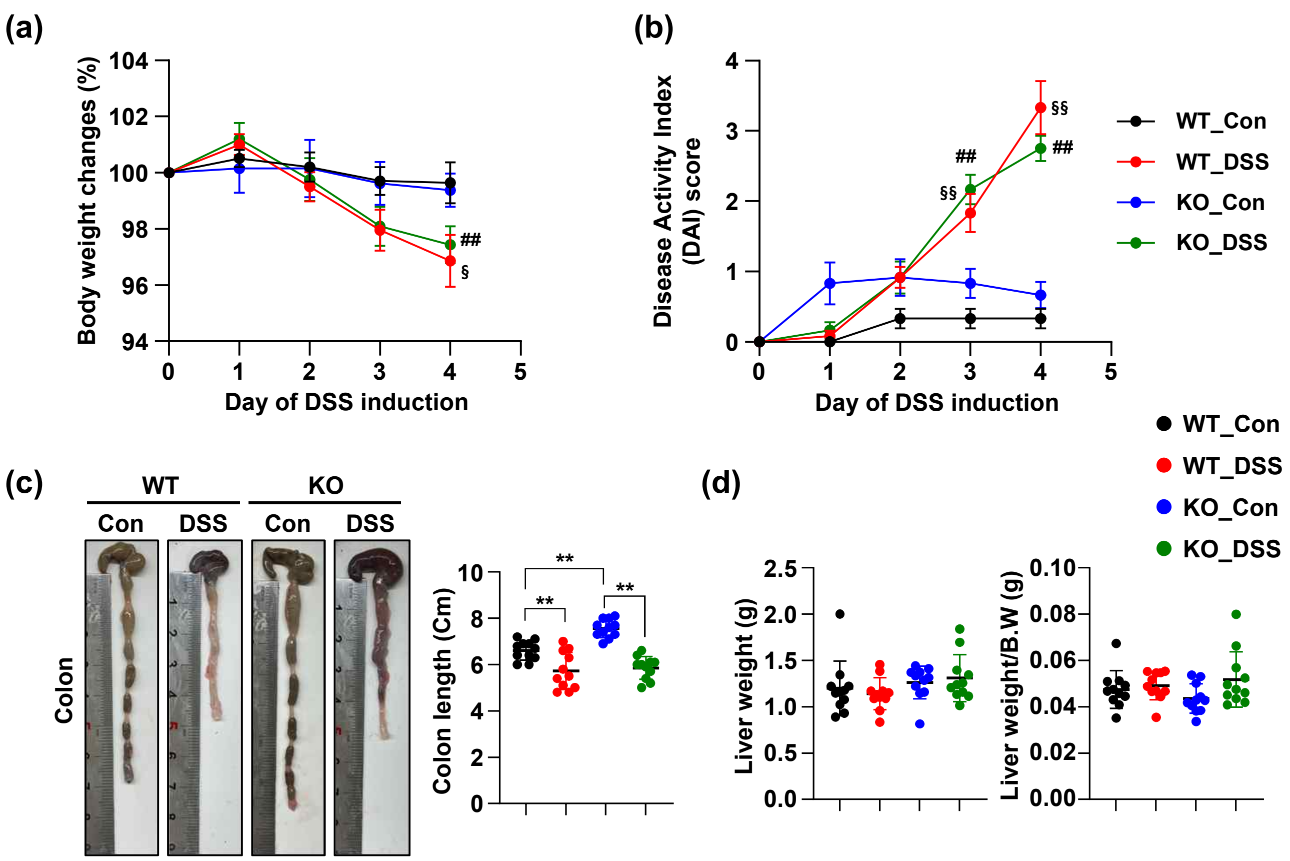

Figure S2. Effects of TLR5 deficiency on DSS-induced colitis in mice.

The colitis was induced by treatment with 2% DSS. WT and TLR5 KO mice were administered 2% DSS in drinking water for four days and distilled water for the following four days (n = 11). (a) Mouse body weights are measured daily. (b) Calculated DAI scores. (c) Colon images and lengths in control and DSS-induced WT and TLR5 KO mice. (d) Liver and liver weight/B.W of control and DSS-induced WT and TLR5 KO mice. All values are presented as the mean ± SD. Statistical significance was measured using one-way or two-way ANOVA with the Bonferroni post-test. *$P < 0.05$, **$P < 0.005$. $^{\S}P<0.05$, $^{\S\S}P<0.005$ compared with the WT control group. $^{\#\#}P<0.005$ compared with the TLR5 KO control group.

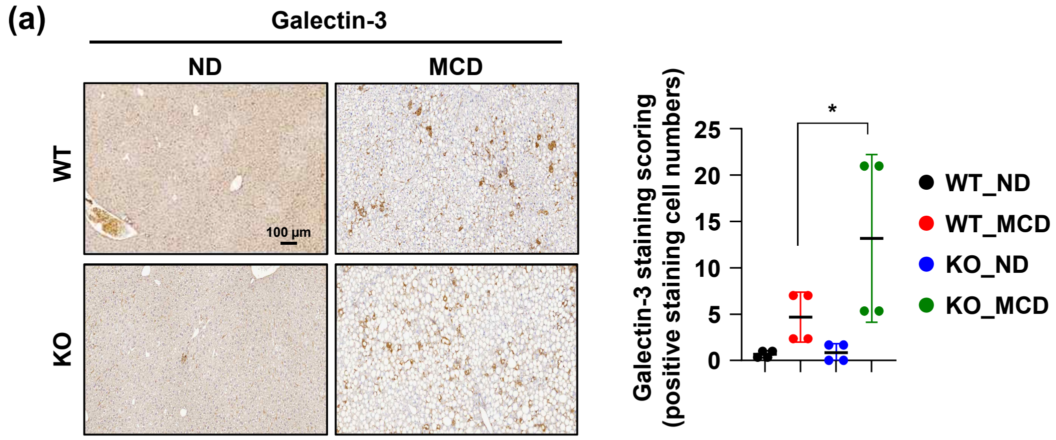

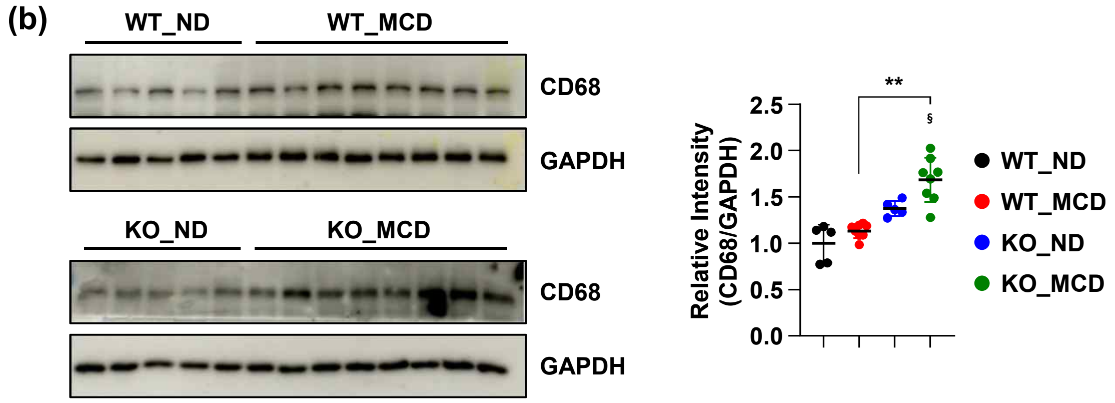

Figure S3. TLR5 deficiency increases fibrosis and inflammation in MCD diet mice.
(a) Immunohistochemical staining of galectin-3 in liver tissue section from normal and two weeks MCD diet-induced WT and TLR5 KO mice. Quantification of positive staining cell numbers of galectin-3 was shown (right panel) (n = 4). (b) Levels of CD68 in liver extracts were determined by western blotting. GAPDH was used as the loading control. The relative protein levels are shown. The values for the normal diet WT group were set to 1 (n = 5 and 8). All values are presented as the mean ± SD. Statistical significance was measured using two-way ANOVA with the Bonferroni post-test. *$P < 0.05$, **$P < 0.005$. §$P<0.05$ compared with the normal diet TLR5 KO group.

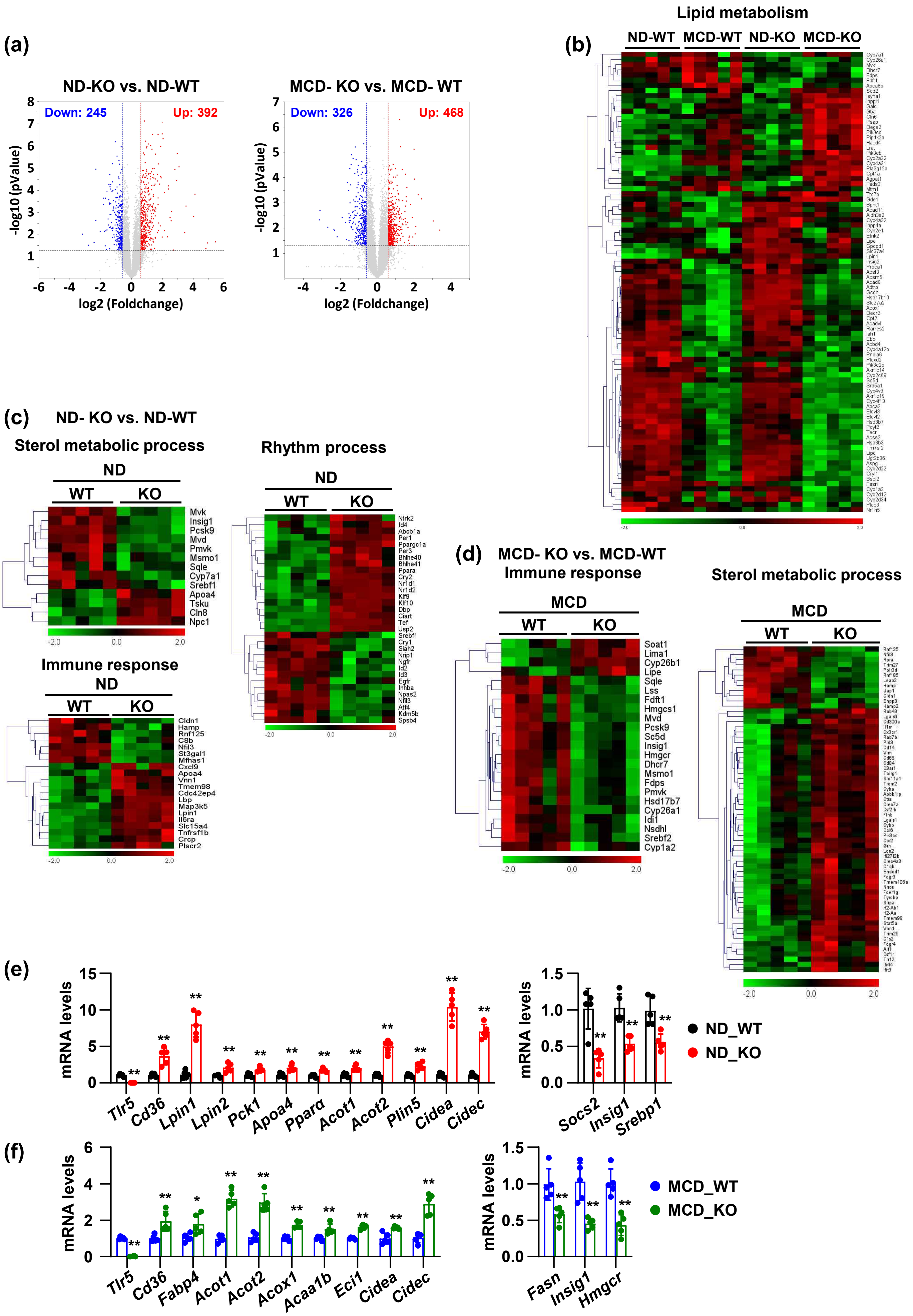

(a)
ND-KO vs. ND-WT
Down: 245
Up: 392
-log10 (pValue)
log2 (Foldchange)
MCD- KO vs. MCD- WT
Down: 326
Up: 468
(b)
Lipid metabolism
ND-WT
MCD-WT
ND-KO
MCD-KO
(c)
ND- KO vs. ND-WT
Sterol metabolic process
ND
WT
KO
Mvk
Insig1
Pcsk9
Mvd
Pmvk
Msmo1
Sqle
Cyp7a1
Srebf1
Apoa4
Tsku
Cln8
Npc1
Rhythm process
Ntrk2
Id4
Abcb1a
Per1
Ppargc1a
Per3
Bhlhe40
Bhlhe41
Ppara
Cry2
Nr1d1
Nr1d2
Klf9
Klf10
Dbp
Ciart
Tef
Usp2
Srebf1
Cry1
Siah2
Nrip1
Ngfr
Id2
Id3
Egfr
Inhba
Npas2
Nfil3
Atf4
Kdm5b
Spsb4
Immune response
Cldn1
Hamp
Rnf125
C8b
Nfil3
St3gal1
Mfhas1
Cxcl9
Apoa4
Vnn1
Tmem98
Cdc42ep4
Lbp
Map3k5
Lpin1
Il6ra
Slc15a4
Tnfrsf1b
Crcp
Plscr2
(d)
MCD- KO vs. MCD-WT
Immune response
Sterol metabolic process
MCD
WT
KO
Soat1
Lima1
Cyp26b1
Lipe
Sqle
Lss
Fdft1
Hmgcs1
Mvd
Pcsk9
Sc5d
Insig1
Hmgcr
Dhcr7
Msmo1
Fdps
Pmvk
Hsd17b7
Cyp26a1
Idi1
Nsdhl
Srebf2
Cyp1a2
-2.0
0.0
2.0
(e)
mRNA levels
Tlr5
Cd36
Lpin1
Lpin2
Pck1
Apoa4
Pparα
Acot1
Acot2
Plin5
Cidea
Cidec
Socs2
Insig1
Srebp1
ND_WT
ND_KO
(f)
mRNA levels
Tlr5
Cd36
Fabp4
Acot1
Acot2
Acox1
Acaa1b
Eci1
Cidea
Cidec
Fasn
Insig1
Hmgcr
MCD_WT
MCD_KO

(g)

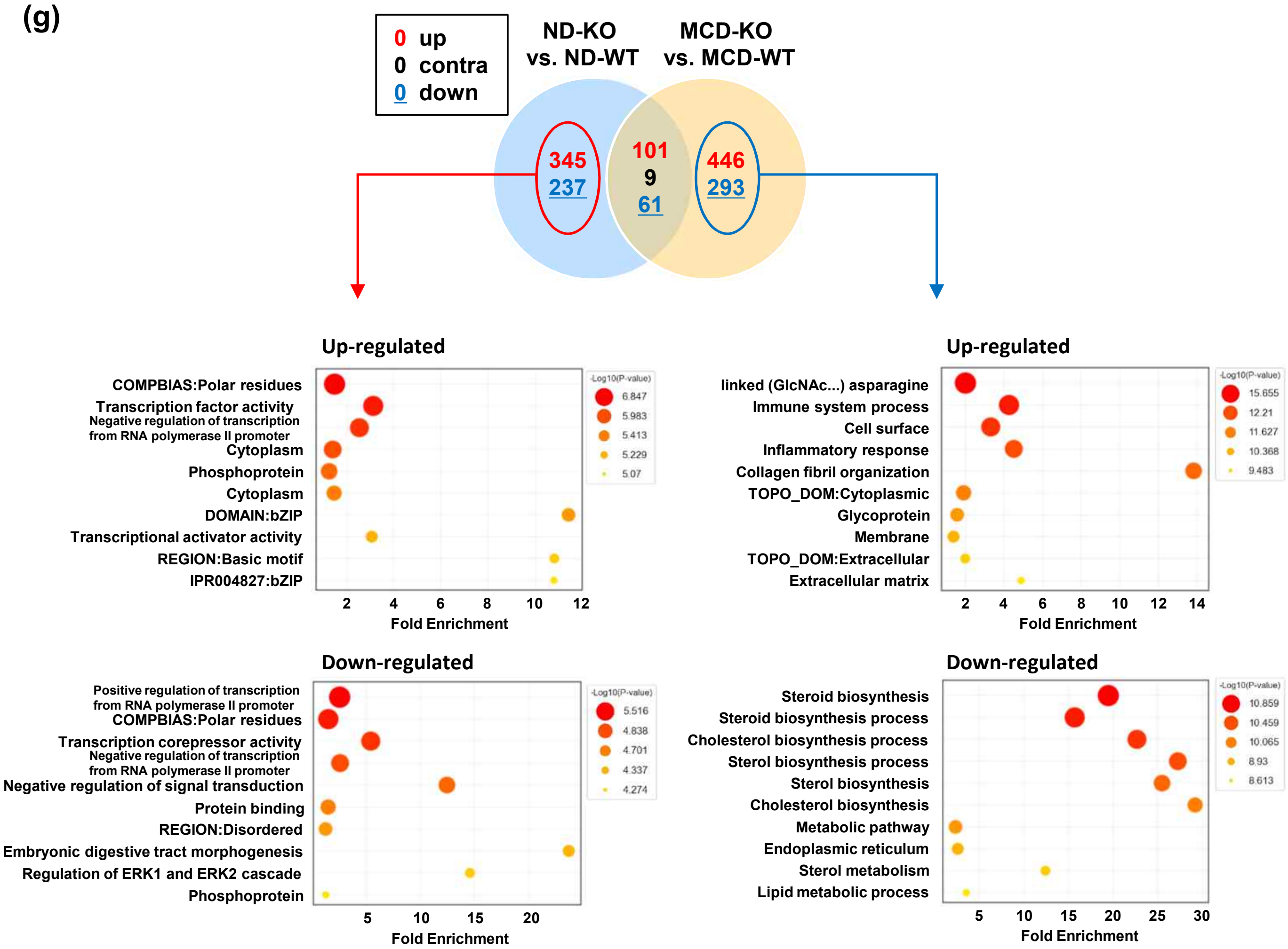

Figure S4. RNA-Sequencing analysis of normal and MCD diet WT and TLR5 KO mice liver.

(a) Volcano plot of RNA-seq data obtained using normal diet (ND) and MCD diet WT and TLR5 KO liver tissues. Blue, down-regulated genes; red, up-regulated genes. (b-d) Hierarchical clustering shows changes in gene expression in lipid metabolism related genes of all groups (b), ND TLR5 KO compared with ND WT mice (c), and MCD diet TLR5 KO compared with MCD diet WT mice (d). (e and f) The mRNA levels of the indicated genes in WT and TLR5 KO livers of ND (e) and two weeks MCD diet (f) were measured by qRT-PCR (n = 5 mice per group). (g) Venn diagram of genes changed in ND-KO vs. ND-WT and MCD-KO vs. MCD-WT. GO analysis of genes showing up- and down-regulated expression. All values are presented as the mean ± SD. Statistical significance was measured using two-way ANOVA with the Bonferroni post-test. $^{*}P < 0.05$, $^{**}P < 0.005$.

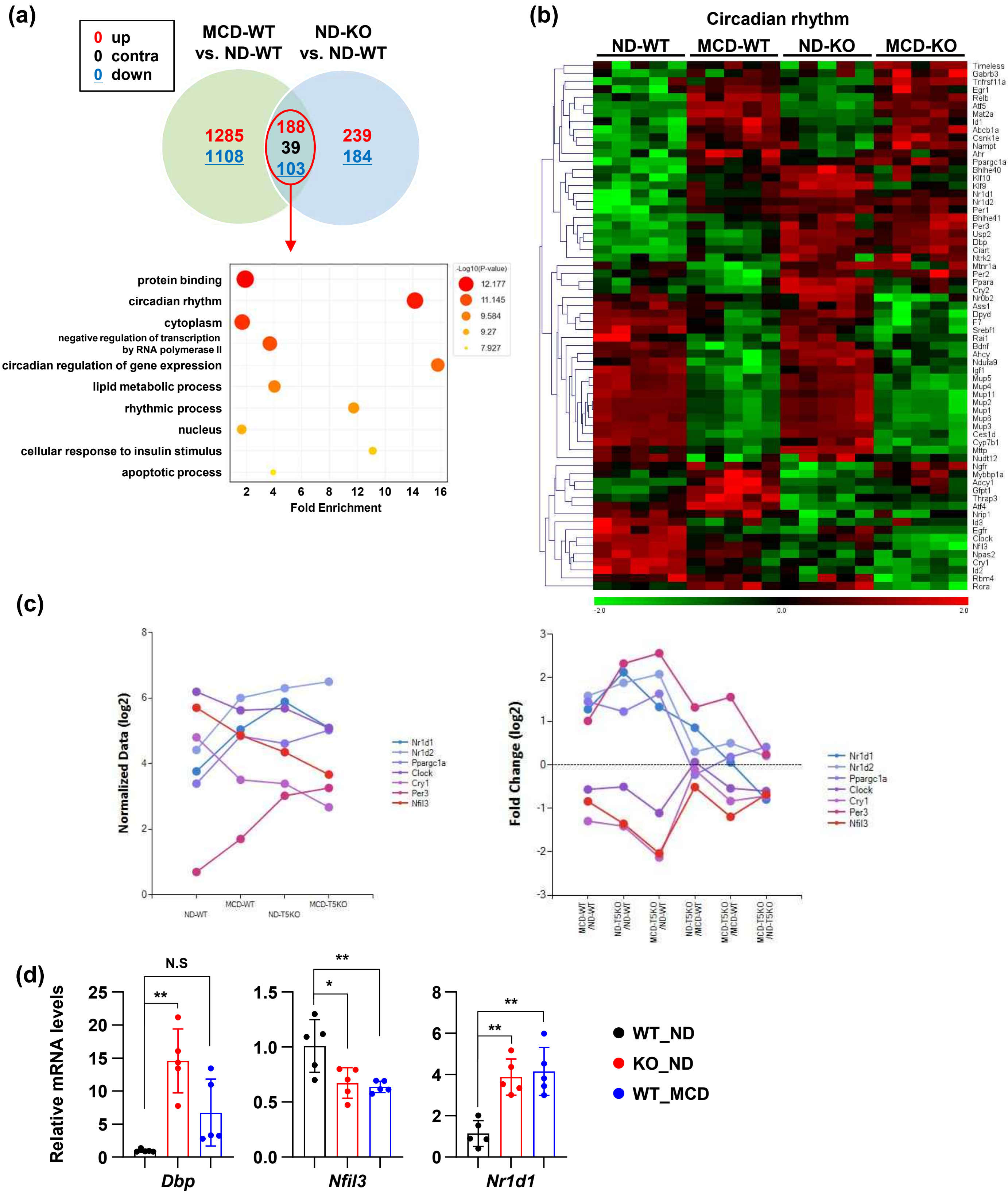

(a)
0 up
0 contra
0 down
MCD-WT vs. ND-WT
ND-KO vs. ND-WT
1285
1108
188
39
103
239
184
protein binding
circadian rhythm
cytoplasm
negative regulation of transcription by RNA polymerase II
circadian regulation of gene expression
lipid metabolic process
rhythmic process
nucleus
cellular response to insulin stimulus
apoptotic process
Fold Enrichment
-Log10(P-value)
12.177
11.145
9.584
9.27
7.927
(b)
Circadian rhythm
ND-WT
MCD-WT
ND-KO
MCD-KO
Timeless
Gabrb3
Tnfrsf11a
Egr1
Relb
Atf5
Mat2a
Id1
Abcb1a
Csnk1e
Nampt
Ahr
Ppargc1a
Bhlhe40
Klf10
Klf9
Nr1d1
Nr1d2
Per1
Bhlhe41
Per3
Usp2
Dbp
Ciart
Ntrk2
Mtnr1a
Per2
Ppara
Cry2
Nr0b2
Ass1
Dpyd
F7
Srebf1
Rai1
Bdnf
Ahcy
Ndufa9
Igf1
Mup5
Mup4
Mup11
Mup2
Mup1
Mup6
Mup3
Ces1d
Cyp7b1
Mttp
Nudt12
Ngfr
Mybbp1a
Adcy1
Gfpt1
Thrap3
Atf4
Nrip1
Id3
Egfr
Clock
Nfil3
Npas2
Cry1
Id2
Rbm4
Rora
-2.0
0.0
2.0
(c)
Normalized Data (log2)
ND-WT
MCD-WT
ND-T5KO
MCD-T5KO
Nr1d1
Nr1d2
Ppargc1a
Clock
Cry1
Per3
Nfil3
Fold Change (log2)
MCD-WT /ND-WT
ND-T5KO /ND-WT
MCD-T5KO /ND-WT
ND-T5KO /MCD-WT
MCD-T5KO /MCD-WT
MCD-T5KO /ND-T5KO
(d)
Relative mRNA levels
N.S
**
*
Dbp
Nfil3
Nr1d1
WT_ND
KO_ND
WT_MCD

(e) **MCD-WT vs. ND-WT**

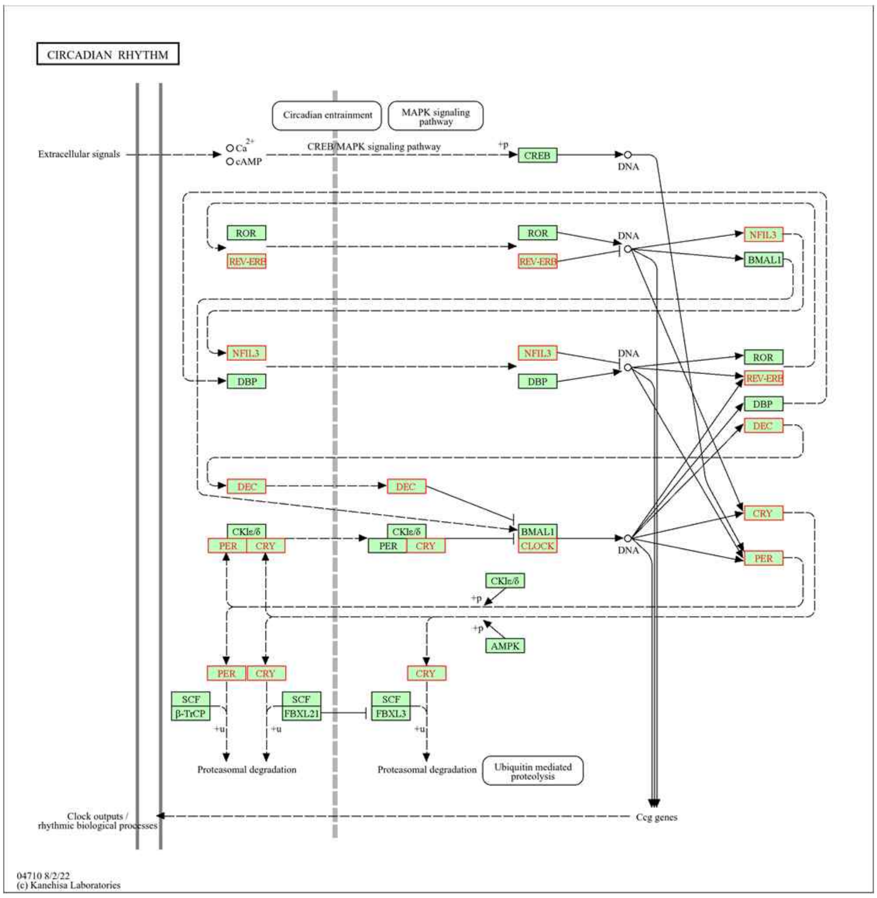

(f) **ND-KO vs. ND-WT**

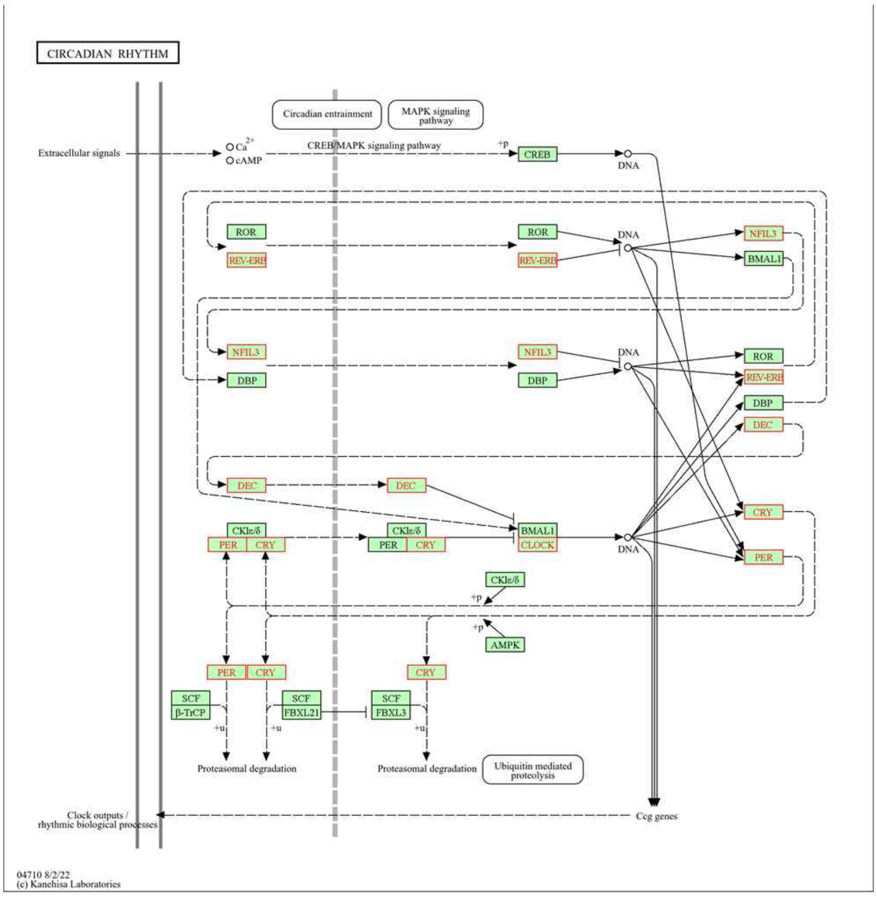

Figure S5. Changes in circadian rhythm-related genes by MCD diet and TLR5 deficiency. (a) Venn diagram of genes changed in MCD-WT vs. ND-WT and ND-KO vs. ND-WT. GO analysis of genes showing up- and down-regulated expression in the overlapped genes (bottom). (b) Hierarchical clustering shows changes in circadian rhythm-related gene expression in all groups. (c) Expression plots for normalized and fold change of the indicated genes. (d) The mRNA levels of the indicated genes in ND-WT, ND-KO, and MCD-WT were measured by qRT-PCR (n = 5 mice per group). (e and f) Expression profiles of circadian rhythm-related genes visualized on a KEGG (Kyoto Encyclopedia of Genes and Genomes) pathway diagram in MCD-WT vs. ND-WT (e) and ND-KO vs. ND-WT (f). All values are presented as the mean ± SD. Statistical significance was measured using two-way ANOVA with the Bonferroni post-test. *P < 0.05, **P < 0.005. N.S. is statistically not significant.

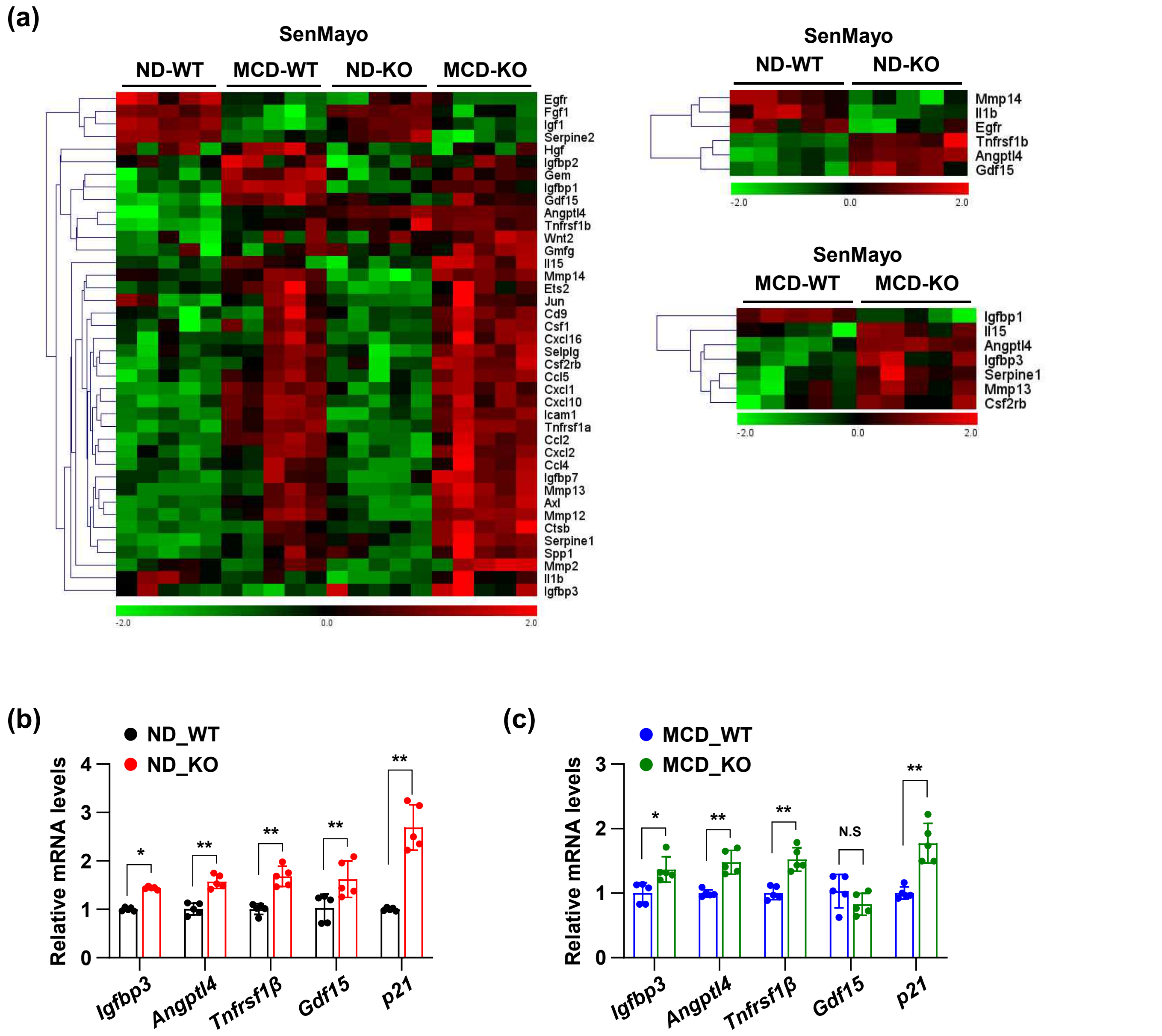

Figure S6. Changes in senescence-related genes by MCD diet and TLR5 deficiency.

(a) Hierarchical clustering shows changes in gene expression in senescence-related genes (SenMayo) of all groups, ND TLR5 KO compared with ND WT mice, and MCD diet TLR5 KO compared with MCD diet WT mice. (b and c) The mRNA levels of the indicated genes in WT and TLR5 KO livers of ND (b) and two weeks MCD diet (c) were measured by qRT-PCR (n = 5 mice per group). All values are presented as the mean ± SD. Statistical significance was measured using two-way ANOVA with the Bonferroni post-test. *P < 0.05, **P < 0.005.

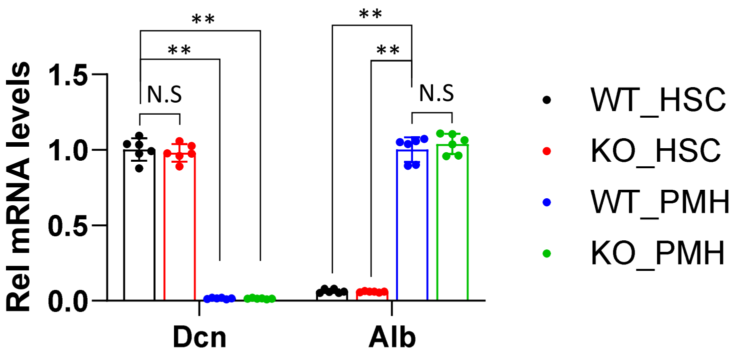

Figure S7. Purity of isolated PMHs and HSCs from mouse liver.

The mRNA levels of the indicated genes in PMHs and HSCs isolated from 22-week-old WT and TLR5 KO mice were measured by qRT-PCR (n = 3 per group). All values are presented as the mean ± SD. Statistical significance was measured using two-way ANOVA with the Bonferroni post-test. *P < 0.05, **P < 0.005.
