# Supplementary material for: The Impact of Toll‐Like Receptor 5 on Liver Function in Age‐Related Metabolic Disorders
Source: Aging Cell. 2025 Feb 17;24(6):e70009. doi: 10.1111/acel.70009 (PMC12151890; doi:10.1111/acel.70009)
Supplement: Supplementary file 3 — Table S1. [file ACEL-24-e70009-s004.pdf]

Table S1. Mouse qPCR primer seqeunces		
Gene	Forward	Reverse
Tlr5	AACATCATCCAGGGAAGCCA	GGAGAAGATAAAGCCGTGCG
Cd36	CTGGGACCATTGGTGATGAAA	CACCACTCCAATCCCAAGTAAG
Scd1	GCAAGCTCTACACCTGCCTCTT	CGTGCCTTGTAAGTTCTGTGGC
Pparγ	GATGGAAGACCACTCGCATT	AACCATTGGGTCAGCTCTTG
Plin5	CACCTTTGCTGATGCACACT	TCCAGGAACTCATCCACACA
Pck1	AAGGAAAACGCCTTGAACCT	GTAAGGGAGGTCGGTGTTGA
Col1a1	GAGCGGAGAGTACTGGATCG	AGACGGCTGAGTAGGGAACA
Col3a1	ACCAAAAGGTGATGCTGGAC	GACCTCGTGCTCCAGTTAGC
Fasn	CCTGGATAGCATTCCGAACCT	AGCACATCTCGAAGGCTACACA
Srebp1c	GCAGCCACCATCTAGCCTG	CAGCAGTGAGTCTGCCTTGAT
Lpin1	TGCAGTTTGTGAACGAGGAG	TGGAAGGGGAATCTGTCTTG
Slc27a1	AAGGTCAATGAGGACACGATG	ATCTTCTTGTTGGTGGCACTG
G6pc	CCTTGTGTCTGTGATTGCTGA	CCACTTGAAGACGAGGTTGAA
Fbp1	TCAACTGCTTCATGCTGGAC	GGGTCAAAGTCCTTGGCATA
Fibronectin	ACAGAGCTCAACCTCCCTGA	TGTGCTCTCCTGGTTCTCCT
Bambi	TGCTGCTCATTATGTTGGCC	CCATGCACTCCAAGTCCAAC
Pdgfrβ	ATCGCGCCACCTTAATCAAC	CAGATCTTGACCAGCTTGCC
Timp1	ACCTATAGTGCTGGCTGTGG	AAGTGACGGCTCTGGTAGTC
α-Sma	CTGACAGAGGCACCACTGAA	AGAGGCATAGAGGGACAGCA
Apoa4	ATGCCAAGGAGGCTGTAGAA	CCCACTCAGCTGTACGACAA
Pparα	TTCACGATGCTGTCCTCCTT	GATGTCACAGAACGGCTTCC
Acot1	CAACTACGATGACCTCCCCA	AATCCCAAGCAGCCCAATTC
Acot2	GCTATGGCCTCCTTCCTGAA	AGCTTCCACGACATCCAAGA
Cidea	TATGTCCCAGTCTGCAAGCA	ACATTGAGACAGCCGAGGAA
Cidec	AGCTAGCCCTTTCCCAGAAG	GTGCAGGTCATAGGAAAGCG
Socs2	CGACTAACCTGCGGATTGAG	GTGAACAGTCCCATTCCGTG
Insig1	CTTTGGGTTTGTGGTGGACA	GGGGAGCCAAGAACGGATAT
Fabp4	TTTCCTTCAAACTGGGCGTG	CATTCCACCACCAGCTTGTC
Acox1	TTGGAAACCACTGCCACATA	AGGCATGTAACCCGTAGCAC
Acaa1b	TTCTCCAGGACGTGAAGCTAA	GTCCCGATGAACACTGTCTGT
Eci1	GGGGTTGCAGTGATGAAGTT	CCTCGGATGCTCTTGTCATT
Hmgcr	AGTGGGAACTATTGCACCGA	ACACCTCTCTCACCACCTTG
Dbp	GGCCATGAGACTTTTGACCC	CCGGCTCCAGTACTTCTCAT
Nfil3	ATGAGGGTGTAGTGGGCAAG	GTTCACTTCCGGAACCTTCA
Nr1d1	ATGCCCATGACAAGTTAGGC	GCCAAAAGGTTGTTGTCGTT
Igfbp3	TTCCATCCACTCCATGCCAA	AGTTCTGGGTGTCTGTGCTT
Angptl4	GATGGAGGCTGGACAGTGAT	CTTCCTCGGTTCCCTGTGAT
Tnfrsf1β	GCCAAACTCCAAGCATCCTT	AACATCAGCAGACCCAGTGA
Gdf15	AACTGAGGTTCCTGCTGTTCC	TGGTTCTGAGTTCGAGTCCTC
p21	ACAAGAGGCCCAGTACTTCC	GTTTTCGGCCCTGAGATGTT
Dcn	CAACAAACTCCTCAGGGTGC	AGGGTTGCCGTAAAGACTCA
Alb	TGAGCCAGACATTCCCCAAT	GTCGCCTGGTTTTCACACAT
GAPDH	TCTGACGTGCCGCCTGGAGA	CAGCCCGGCATCGAAGGTG
